# Systems Pharmacology and Microbiome Dissection of Shen Ling Bai Zhu San Reveal Multiscale Treatment Strategy for IBD

**DOI:** 10.1155/2019/8194804

**Published:** 2019-06-23

**Authors:** Wei-jie Lv, Cui Liu, Yue-fei Li, Wen-qian Chen, Zeng-quan Li, Yue Li, Ying Xiong, Li-min Chao, Xiao-long Xu, Shi-ning Guo

**Affiliations:** ^1^College of Veterinary Medicine, South China Agricultural University, Guangzhou 510642, China; ^2^Beijing Hospital of Traditional Chinese Medicine, Affiliated with Capital Medical University, Beijing 100010, China; ^3^Guangdong Research Center for Veterinary Traditional Chinese Medicine and Natural Medicine Engineering Technology, Guangzhou 510642, China

## Abstract

Generally, inflammatory bowel disease (IBD) can be caused by psychology, genes, environment, and gut microbiota. Therefore, IBD therapy should be improved to utilize multiple strategies. Shen Ling Bai Zhu San (SLBZS) adheres to the aim of combating complex diseases from an integrative and holistic perspective, which is effective for IBD therapy. Herein, a systems pharmacology and microbiota approach was developed for these molecular mechanisms exemplified by SLBZS. First, by systematic absorption-distribution-metabolism-excretion (ADME) analysis, potential active compounds and their corresponding direct targets were retrieved. Then, the network relationships among the active compounds, targets, and disease were built to deduce the pharmacological actions of the drug. Finally, an “IBD pathway” consisting of several regulatory modules was proposed to dissect the therapeutic effects of SLBZS. In addition, the effects of SLBZS on gut microbiota were evaluated through analysis of the V3-V4 region and multivariate statistical methods. SLBZS significantly shifted the gut microbiota structure in a rat model. Taken together, we found that SLBZS has multidimensionality in the regulation of IBD-related physiological processes, which provides new sights into herbal medicine for the treatment of IBD.

## 1. Introduction

Recent studies have revealed several factors responsible for the digestive diseases such as irritable bowel syndrome/inflammatory bowel disease (IBS and IBD) [1–5]. However, the cellular mechanisms behind these diseases are complex and unclear. In recent years, much attention has been focused on the development of herbal medicine for the treatment of digestive diseases. Shen Ling Bai Zhu San (SLBZS), which is composed of 10 herbs, has been proven to have wide pharmacological effects on digestive diseases, including anti-inflammatory and gut microbiota modulation effect [6, 7].

A majority of herbal medicines exert pharmacological effects by targeting multiple host molecules. However, it is difficult to identify these herbal medicine targets. Therefore, a new method that can identify the active compounds and pharmacological targets of herbal medicine is in urgent need of development [8].

Systems pharmacology, which combines oral bioavailability prediction, multitarget prediction, and network analyses, is used to identify the active compounds and pharmacological targets of herbal medicine [9–11]. Herein, we applied the systems pharmacology method to explore the pharmacological mechanisms of SLBZS.

Additionally, high-throughput sequencing has been used to promote our understanding of the role of gut microbiota in health and disease [12]. For example, small intestinal bacterial overgrowth [13] and altered intestinal microbiota [14] are implicated in subgroups of patients with functional bowel disorders. However, due to the inherent limitations of the diagnostic methods, the exact evidence of the causal role of microbiota composition on the pathogenesis of the disease remains elusive.

In this study, a combination of systems pharmacology and 16S rRNA boosts our exploration of the potential relationship among drug-microbiota-target.

## 2. Results

In this study, a combination of oral bioavailability (OB) (≥30%) screening, Caco-2 permeability (Caco-2) (>-0.4), prediction of permeability, half-life (HL) (long), and drug-likeness (DL) (≥0.18) properties was applied to explore the active compounds of SLBZS. We also added DL < 0.18, OB < 30%, Caco − 2≤−0.4, and HL = short with bioactivity as candidate compounds. Finally, we screened 97 SLBZS as candidate compounds (Table 1). The number of active compounds in *Dolicho LablabL*, *Atractylodes macrocephala*, *Wolfiporia cocos*, *Glycyrrhiza uralensis Fisch*, *Platycodon grandiflorus*, *Semen Nelumbinis*, *Panax Ginseng*, *Fructus Amomi*, *Dioscorea opposita*, and Semen *Coicis* was 4, 7, 15, 20, 3, 8, 23, 9, 10, and 8, respectively.

### 2.1. Target Identification and Network Analysis

SLBZS exerts a pharmacological effect by targeting several host molecules [15, 16]. To identify the drug-target direct interactions on a large scale, we hypothesized that the ensemble features of the ligand group can accurately reflect the direct binding information of a specific target based on ligand-target interaction data to establish the WES model. In the present study, 74 potential targets were predicted for the 97 candidate compounds (Supp Table S1 and S2).

### 2.2. Network Construction

#### 2.2.1. Compound-Target Network

**Figure 1:**
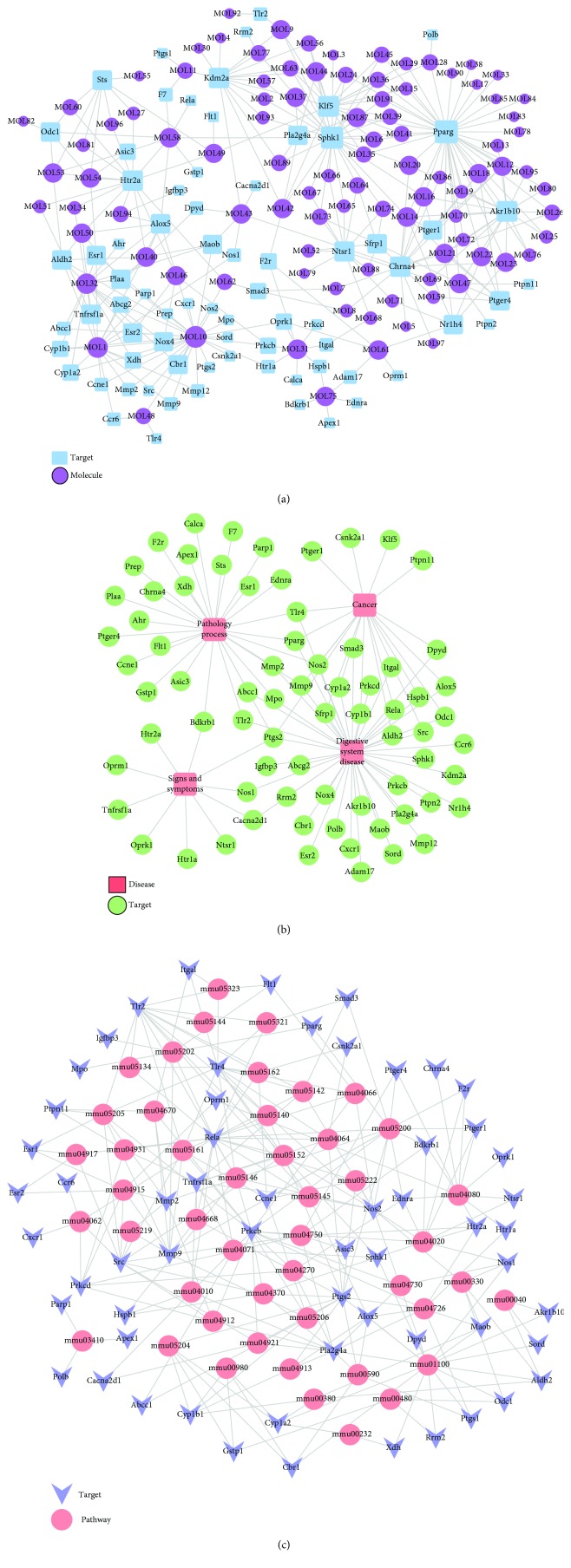
Compound-target-disease-pathway network. (a) Compound-target network of SLBZS consisting of 171 nodes (97 compounds and 74 potential targets) and 330 edges. (b) Target-disease network including 73 candidate targets and 4 diseases. (c) Target-pathway network including 59 candidate targets and 47 KEGG pathways.

The compounds from SLBZS acted on multiple targets, and each target was involved with a variety of compounds.  Figure 1(a) shows that the compound-target network contains 171 nodes and 330 compound-target interactions. We screened 74 target proteins from the potential target list related to intestinal disease processes, including inflammatory bowel disease, duodenal ulcer, and colitis. Additionally, multiple relationships between the compounds and targets were illustrated in this network. For instance, quercetin exhibited the highest number of target candidate-target interactions (degree = 28), followed by luteolin (degree = 7) and kaempferol (degree = 15), thereby indicating the multitarget properties of SLBZS ingredients.

#### 2.2.2. Target-Disease Network

To identify the relationship between SLBZS and disease, the DrugBank, TTD, and PharmGKB databases were searched. As shown in Figure 1(b) and Supp Table S3, 73 SLBZS compounds were related to 4 types of diseases, including digestive system disease (degree = 44), pathology processes (degree = 28), signs and symptoms (degree = 10), and cancer (degree = 19).

#### 2.2.3. Target-Pathway Network

**Figure 2:**
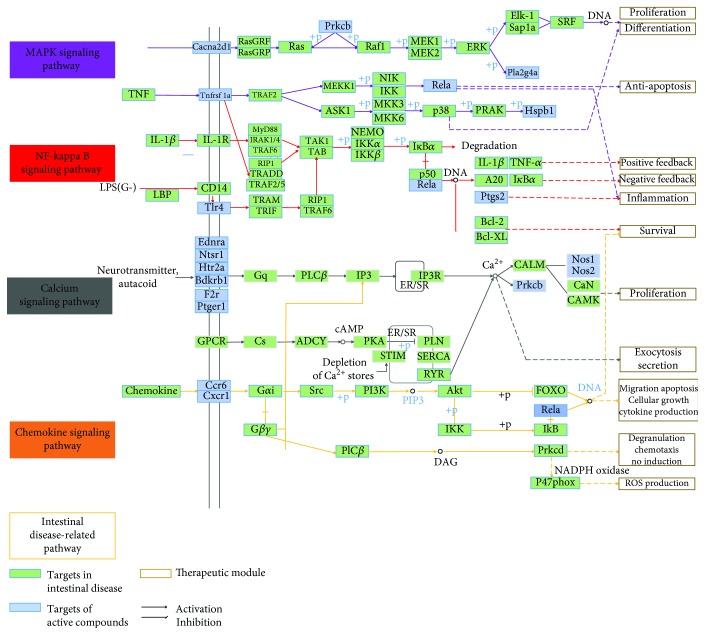
Intestinal disease-related pathway including MAPK signaling pathway, NF-kappa B signaling pathway, calcium signaling pathway, and chemokine signaling pathway.

A total of 59 candidate targets and 47 KEGG pathways constituted the target-pathway network (Figure 1(c)). Apparently, most targets were related to different pathways, indicating that SLBZS may exert synergistic effects through these different pathways. Additionally, one active compound may target different molecular targets, illustrating the multitarget mechanism of SLBZS. Moreover, to investigate the synergic effects of these 10 herbal medicines on IBD, an integrated “digestive-related pathway” approach was applied based on the current research of digestive disease pathology including the MAPK signaling pathway, NF-kappa B signaling pathway, calcium signaling pathway, and chemokine signaling pathway (Figure 2 and Supp Table S4 and S5).

#### 2.2.4. Target Tissue Location

Supp Figure S1 shows the tissue location network based on these 70 targets, which can be divided into six tissue modules, including the liver, heart, spleen, lung, kidney, and gut. Thus, the candidate compounds reflect multiple targets that can be localized to more than one tissue (Supp Table S6).

### 2.3. Structural Changes of Intestinal Microbiota by SLBZS

**Figure 3:**
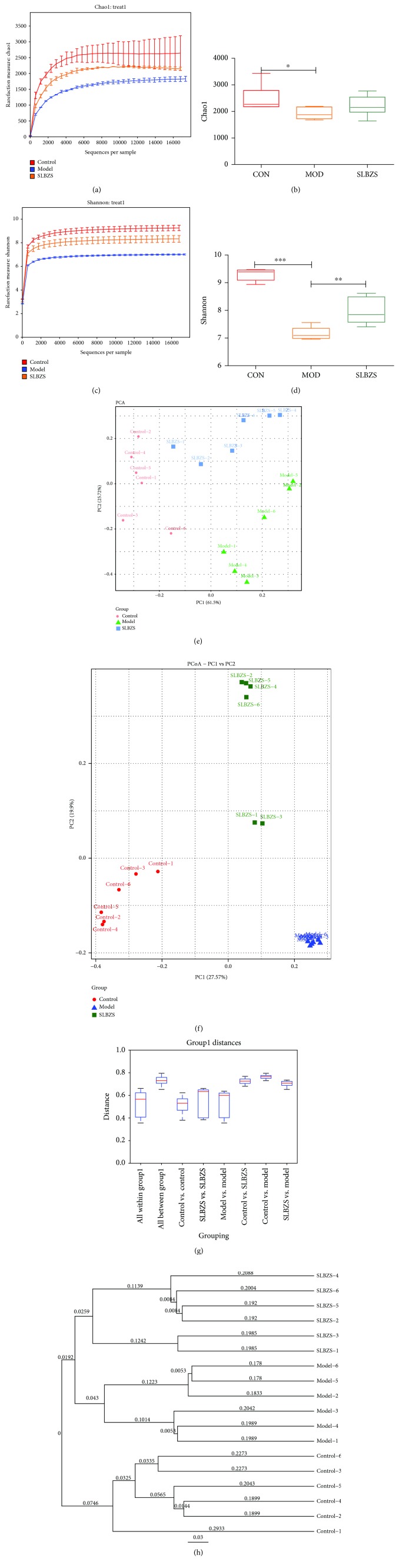
The effect on the gut microbiota structure of SLBZS. (a–d) Rarefaction curves showing microbial richness based on the Chao1 index and microbial richness and evenness on the Shannon index. (e, f) Microbiome clustering based on unweighted principal component analysis (PCA) and principal coordinate analysis (PCoA) UniFrac metrics of fecal gut microbiota. (g, h) Unweighted UniFrac distance and unweighted pair-group method with arithmetic means (UPGMA) showed that DSS and SLBZS treatment can separate rats clearly. Statistical significant difference was assessed through one-way ANOVA with LSD post hoc test ^∗^ *P* < 0.05, ^∗∗^ *P* < 0.01, and ^∗∗∗^ *P* < 0.001; *n* = 6.

To analyze the alteration in the intestinal microbiota structure in rats treated with herbal medicine for IBD, the Illumina sequencing system was used to generate high-quality sequences from stool samples. The *α* diversity of the intestinal microflora indicates that DSS can significantly decrease the Chao1 and Shannon diversity indices in both experimental groups (MOD and SLBZS), whereas the SLBZS group showed a higher Shannon index when compared to the Chao1 index after treatment with SLBZS (Figures 3(a)–3(d)). Principal component analysis (PCA) and principal coordinate analysis (PCoA) showed that SLBZS significantly altered the intestinal microbiota structure of rats (Figures 3(e) and 3(f)). Similarly, the unweighted UniFrac distance and unweighted pair-group method with arithmetic means (UPGMA) showed that DSS and SLBZS treatment can clearly separate rats (Figures 3(g) and 3(h)).

**Figure 4:**
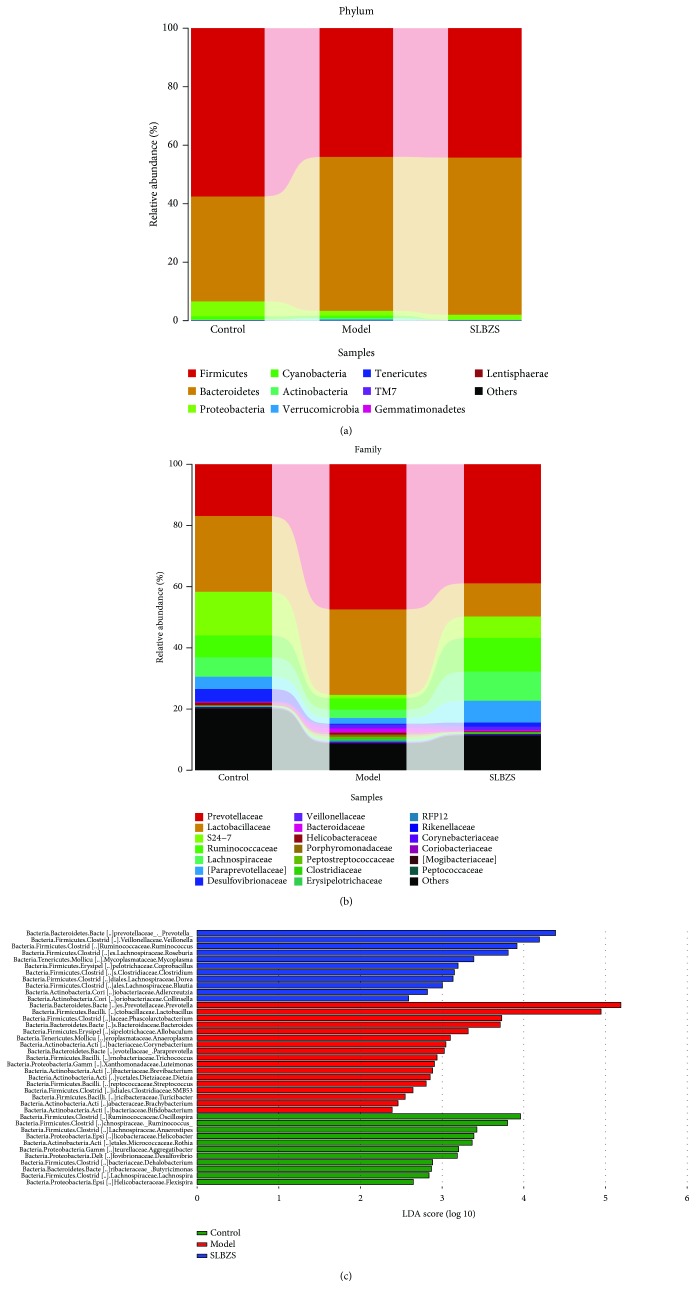
Gut microbiota analysis upon SLBZS treatment in colitic rats. (a, b) Bar plots of the taxonomic composition showing relative abundances > 1% of bacterial phyla (a) and families (b). (c) Comparison of the relative abundances of different taxa among control, model, and SLBZS-treated rats.

To find key system types related to the efficacy of SLBZS, MetaStat analysis was used in this study. Using mothur software, the statistical algorithm of MetaStat [17] was used to perform a pairwise comparison test on the differences between the samples (groups) of each taxon at the gate and genus levels. A detailed phylogenetic analysis of the taxonomic composition of the microbiome of mice treated with DSS showed that the increased inflammatory conditions observed upon DSS administration were associated with variations in the abundance of specific taxa, including *Firmicutes*, *Bacteroidetes*, *Proteobacteria*, and *Actinobacteria* [18, 19] (Figure 4(a)).

Significant changes towards restoration of normobiosis were detected among the less abundant genera belonging to the *Firmicutes* phylum in the DSS+SLBZS-derived microbiota. For instance, *Corynebacteriaceae*, *Lactobacillaceae*, *Paraprevotellaceae*, *Veillonellaceae*, *Prevotellaceae*, and *Clostridiaceae* which were expanded in colitic rats were reduced upon SLBZS treatment and returned to levels comparable to those observed in control group rats (Figure 4(b)).

**Figure 5:**
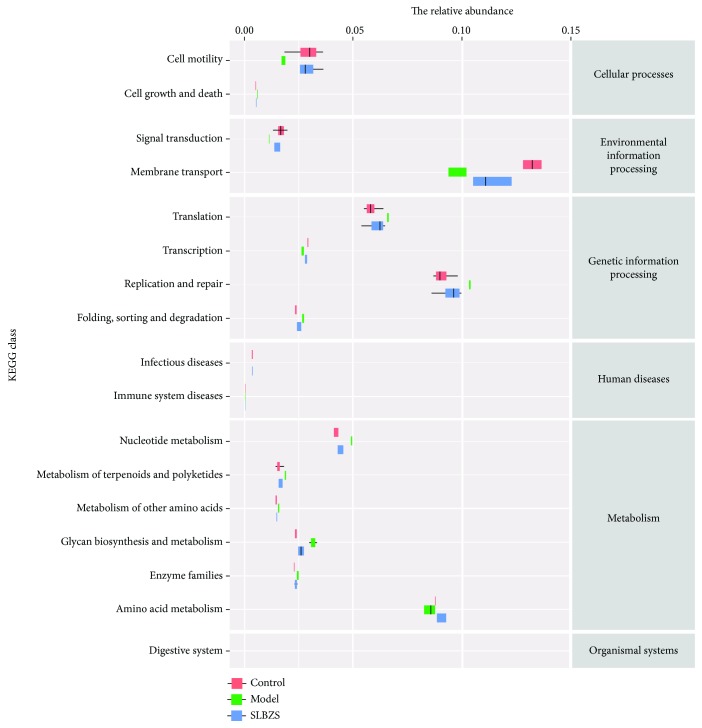
KEGG statistics for the functional genes. The horizontal coordinates represent the relative abundance of functional genes in different groups; the vertical coordinates represent the functions of genes. The relative abundance of functional genes in the following categories was significantly changed (*P* < 0.01).

SLBZS-derived samples showed significant increases in known commensals, including *Adlercreutzia* and *Dorea*, and in the SCFA-producing taxa *Ruminococcus* spp., *Blautia sp.*, *Clostridium spp*., *Veillonella spp*., *Coprococcus* spp., and *Roseburia spp*. (Figure 4(c) and Supp Figure S2), which are reported to be reduced in IBD patients [20–22]. Additionally, PICRUSt was applied to predict microbiota metabolism [23]. 328 pathways were involved in the microbiota metabolism and 176 pathways were significantly changed by SLBZS (Figure 5 and Supp Figure S3). After treatment with SLBZS, the relative abundance of functional genes in the following categories significantly (*P* < 0.01) increased: cell motility, signal transduction, membrane transport, and amino acid metabolism, all of which were reduced in the DSS-treated group. Meanwhile, cell growth and death, replication and repair, infectious diseases, immune system diseases, glycan biosynthesis and metabolism, and digestive system genes were significantly (*P* < 0.01) decreased but were increased in the DSS group.

### 2.4. Alleviation of IL-1*β*, IL-10, and TNF-*α* and Pathological Changes by SLBZS

**Figure 6:**
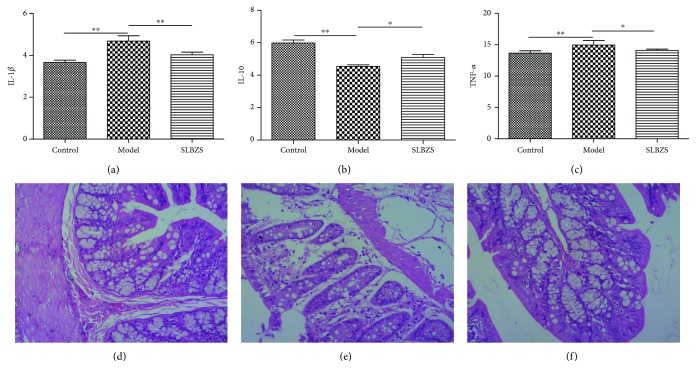
IL-1*β* (a), IL-10 (b), and TNF-*α* (c) can be significantly improved by SLBZS. ^∗^ *P* < 0.05; ^∗∗^ *P* < 0.01. Histological changes of the colon in the control group (d), the model group (e), and the SLBZS group (f). The colon in the control group (d) presented the normal histological feature. In the model group (e), there was intestinal inflammatory cell infiltration, intestinal villus epithelial cell degeneration, necrosis, and shedding. In the SLBZS group (f), the pathological change was significantly reduced. HE staining (×100).

We analyzed the data from the experiment, and after one week of treatment, IL-1*β* and TNF-*α* were significantly decreased by SLBZS, whereas IL-10 was increased. As shown in Figures 6(a)–6(c), IL-1*β* and TNF-*α* were significantly decreased, while IL-10 increased after treatment with SLBZS. The histological changes in the colons of each group are shown in Figures 6(d)–6(f). The colon of the control group exhibited normal histological features. In the model group, there was infiltration of colon mucosal inflammatory cells, and the intestinal villus epithelial cells were degenerated, necrotic, and shed. Pathological changes were significantly reduced in the SLBZS group.

## 3. Discussion

To our knowledge, this is the first study to evaluate the efficacy of TCM on IBD with a systems pharmacology and microbiota approach. Chinese herbs are a complex system of multicomponent, multitarget, and synergistic effects among its components. This systematic pharmacological technique is used to study TCM active compounds and target identification and identify targets and the relationship between drugs and diseases.

In this study, some herbal medicines may contain the same compound, such as *Glycyrrhiza uralensis Fisch* and *Panax Ginseng*, *Panax Ginseng* and *Fructus Amomi*, and *Panax Ginseng* and *Glycyrrhiza uralensis Fisch*. Among these, *Atractylodes macrocephala*, *Glycyrrhiza uralensis Fisch*, and *Panax Ginseng* have been found to have extensive pharmacological effects, such as antioxidant, immunomodulation, and anti-inflammatory [24–29]. In particular, the low molecular weight *Glycyrrhiza uralensis Fisch* can inhibit tumor cell proliferation to exert anticancer and immunomodulatory effects. They also increase the thymus/spleen index and T lymphocyte population. In addition, *Glycyrrhiza uralensis Fisch* can increase the expression of antitumor factors such as IL2, IL6, and IL7 and reduce the protumor cytokine TNF*α* [24]. Shimato et al. have demonstrated that *Atractylodes macrocephala* can promote the production of G-CSF, clinically used to treat neutropenia and prevent chemotherapy-induced immunodeficiency [27]. Also, *Panax Ginseng* has been demonstrated to regulate multiple types of immune cells including macrophages, NK cells, DCs, T cells, and B cells [30]. Moreover, luteolin and quercetin, which existed widely in *Glycyrrhiza uralensis Fisch*, *Platycodon grandiflorus*, and *Semen Nelumbinis*, have been proven to have broad biological and pharmacological effects [31, 32].

Despite the different numbers of each herbal-related targets, significant target overlap occurred between these herbals. The results indicate that these targets can be regulated by different herbs in SLBZS to play a synergistic role [15, 16]. For example, several compounds including *alexandrin_qt*, *campesterol*, *isofucosterol*, and *panaxadiol* were involved in modulating the activation of peroxisome proliferator-activated receptor gamma (Pparg), which might be ascribed to lipid and glucose metabolism [33, 34].

Based on network construction, the results indicate that most compounds modulate multiple targets to exert their pharmacological effects. For example, *quercetin* exhibits not only antitumor [35] and anticancer [31] activity but also antiproliferative effects [36]. Antiproliferative effects of conjugated metabolites of quercetin have been evaluated against three different cancer cell lines [36]. In addition, *luteolin* presents anti-inflammatory effects, serves as a neuroprotective agent [37], and also exhibits antitumor effect [32, 38]. The antitumor effect of *luteolin* may be transmitted by cMet/Akt/ERK signaling [38]. Furthermore, the results indicated that several targets are hit by multiple ingredients in the compound-target network. Pparg showed the highest degree (degree = 39), along with Sphk1 (Sphingosine kinase 1, degree = 31), Klf5 (Kruppel-like factor 5, degree = 19), and Akr1b10 (Aldo-keto reductase family 1 member B10, degree = 19), demonstrating the potential therapeutic effect of each drug contained in SLBZS for combating intestinal disease via modulation of these relevant proteins. These targets play crucial pathological rules in disease related to cancer [39–42]. AKR1B10 has been shown to be closely related to tumor size and cell metastasis of gastric cancer, and AKR1B10 can be used as a good prognostic indicator for gastric cancer [39]. Deletion of KLF5 can result in a decrease in PI3K/AKT signaling and accumulate HIF1*α* in prostate tumors to promote tumor angiogenesis [40]. Previous research has illustrated that the PPAR*γ* allele may be involved in the development, differentiation, and metastasis of gastric cancer in Turkey [43]. Sphk1 has been shown to be involved in the pathogenesis of human hepatocellular carcinoma (HCC), and DMS can inhibit the effects of Sphk1. Therefore, Sphk1 can be used as a potential target for the treatment of HCC [42].

Reportedly, the proteins Mmp12, Pparg, and Ptgs2 are related to digestive system disease and cancer. Biancheri et al. demonstrated that Mmp12 contributes to the responsiveness of patients with IBD to anti-TNF agents [44] and might be involved in the remodeling of injured gut tissue with respect to the migration, proliferation, and differentiation of endothelial cells [45]. Pparg is involved in the inhibition of tissue damage associated with immune activation through the inhibition of the NF-*κ*B pathway [46]. Ptgs2 can repair the injured intestinal mucosa and exert a critical role in the pathophysiology of *Salmonella typhimurium*-induced ulcerative colitis [47].

Some pathways, such as the NF-*κ*B pathway [46], MAPK pathway [48, 49], calcium signaling pathway [50], and chemokine signaling pathway [51], have been improved in relation to digestive disease. In addition, these pathways are reflected in several modules such as inflammation, proliferation apoptosis, survival, and proliferation.

The MAPK cascade is a highly conserved module that is involved in various cellular functions, including cell proliferation, differentiation, and migration. A study on the pharmacological approach showed that stevioside exerts antiapoptotic and anti-inflammatory effects through the inhibition of the release of cytokines and the activation of the MAPK signaling pathway [52]. Another pharmacological study showed that berberine might execute an antiapoptotic function by inhibiting the MAPK pathway [53]. In macrophages, carbon monoxide (CO) reduces lipopolysaccharide-induced proinflammatory cytokines effectuated by the MAPK pathway [48]. Recent studies have highlighted that oxidative stress activates MAPKs and MT2A (a mediator of MAPKs) that play a crucial role in antiapoptosis and anti-inflammation [54]. Studies have shown that black raspberries (BRB) have a preventive effect on rat esophageal cancer, and the mechanism may be that BRB reverse oxidative stress and inhibit NF-*κ*B/MAPK pathway [55]. Rutin exerts neuroprotective effects by increasing superoxide dismutase and glutathione peroxidase levels in the peripheral blood and inhibiting the p38 MAPK pathway [56]. This finding reveals the importance of MAPKs in the normal digestive function.

NF-*κ*B is found in almost all animal cell types and is involved in cellular responses to stimuli such as stress, cytokines, free radicals, heavy metals, ultraviolet irradiation, oxidized LDL, and bacterial or viral antigens. NF-*κ*B also regulates the expression of antiapoptotic genes which play an important role in cell survival and play various roles in cell development, proliferation, differentiation, and metabolism [57]. Furthermore, the degree of NF-*κ*B activation induced by LPS is significantly increased in Atg7-deficient intestinal epithelium. This also shows that autophagy can relieve endotoxin-induced inflammatory responses in intestinal endothelial cells, thereby maintaining intestinal homeostasis [58]. Both innate and adaptive immune responses are associated with NF-*κ*B, and the development and maintenance of cells and tissues associated with the immune system are under the control of the NF-*κ*B transcription factor family in multiple pathways. Initially, the role of NF-*κ*B in the thymus was limited to the important role of RelB [59], but it became clearer in the development of medullary thymic epithelial cells [60–62].

Calcium (Ca^2+^) ions are important for cell signaling, and they exert an allosteric regulation of many enzymes and proteins once they enter the cytosol of the cytoplasm. Studies have shown that intracellular Ca^2+^ release is accomplished by Zn^+^ triggering inositol 1,4,5-triphosphate (IP3) [63, 64]. Activation of mitogen-activated protein (MAP) and phosphoinositide 3 (PI3) kinase pathways protects colonic epithelial cells. In experimental colitis, metabolic calcium signaling in colonocytes is induced by zinc-induced receptors [65]. In Caco-2 cell monolayers, DSS increases the intracellular Ca^2+^ concentration and depletes intracellular Ca^2+^ by BAPTA/AM, disrupts tight junctions, and causes barrier dysfunction [66]. These findings reveal the importance of the calcium signaling pathway, which might be instrumental to the success of future human trials of a new strategy to treat digestive disease.

Chemokine receptors are cytokine receptors found on the surface of certain cells that interact with a type of cytokine called a chemokine. Chemokines can activate a range of signaling pathways to mediate their biological effects by binding to G-protein-coupled receptors (GPCRs). Duffy and D6 bind to CXC and CC inflammatory chemokines, respectively [67]. Chemokines can be divided into “inflammatory” chemokines and “steady state” chemokines [68]. Inflammatory chemokines can play important roles in recruiting leukocytes to the site of inflammation, such as neutrophils, monocyte macrophages, dendritic cells (DC), and natural killer (NK), which play key roles in the innate immune response. The results demonstrated that the chemokine signaling pathway can be further divided into modules such as survival, migration, apoptosis, cellular growth, and cytokine production. In the immune system, chemokines are mainly produced and transported by leukocytes and play an important role in the immune system [69]. Chemokines act by binding to chemokine receptors. Activation of chemokine receptors induces proliferation and differentiation of immune cells, and both are essential processes in innate and adaptive immune responses [70].

In summary, these pathways are regulated by different compounds, suggesting that digestive diseases may affect different pathways. In addition, multiple protein targets belong to a variety of signaling pathways, suggesting that certain proteins can simultaneously affect multiple signaling pathways.

In this experiment, IL-1*β*, IL-10, and TNF-*α* were analyzed. IL-1*β* and TNF-*α* participate in the MAPK and NF-*κ*B pathways, and the two pathways share crosstalk. Recent studies have shown that IL-1*β* and TNF-*α* can be proinflammatory cytokines in IBD [71–74], whereas IL-10 has been demonstrated to play a vital role in the control of inflammation and prevention of enteritis [75]. In patients with colitis and inflammatory bowel disease, damaged mitochondria accumulate in macrophages when IL-10 signal is deficient. This leads to abnormal activation of NLRP3 inflammasome and IL-1*β* production [76, 77].

In our research, changes to the intestinal microbial structure by SLBZS were observed. SLBZS treatment inhibits two genera that contain potential pathogens, *Corynebacterium* and *Helicobacter*, which are strongly associated with peptic ulcers, chronic gastritis, duodenitis, and stomach cancer [78]. *Blautia* is a group of bacteria containing various acetate and butyrate producers [79, 80]. This research observed that an increase in the SCFA producer *Blautia* in the SLBZS treatment group was consistent with previous animal studies [81]. *Roseburia* and *Lachnospira*, as SCFA producers, were reported to recover a balanced community after diet treatment in type 2 diabetic patients [82]. *Ruminococcus* is present in the digestive tract of ≥90% of people and is involved in diseases associated with the intestines, such as IBD. A recent study demonstrated the ability of *Ruminococcus* to produce propanol and propionate as the end products of metabolism [83]. These new findings provide us with insights into the specificity of the genera's adaptability to the gut environment and promote our understanding of the role of gut commensals in health and disease.

Based on the 16S rRNA results, we found that SLBZS treatment can alter the microbial structure of the intestine, which has an enhanced effect on the intestinal microbiota richness and diversity in DSS model rats. Therefore, SLBZS seems to offset the structural changes caused by DSS. As a result, the identified bacterial phylotypes in response to SLBZS treatment in DSS rats may be related to the development and improvement of DSS-induced metabolic abnormalities. The overrepresentation of the KEGG gene in the patient's microbiome can reflect the deleterious metabolism of the neurotransmitter pathway and the host gut protection glycosaminoglycan mucin, in contrast to the beneficial counterpart in the control [84]. Our PICRUSt analyses revealed that SLBZS can significantly increase amino acid metabolism, which may indicate SCFA. This is consistent with the increased SCFA-producing genera. Infectious disease, immune system disease, and digestive system disease were all reduced after treatment with SLBZS, which had a positive effect on IBD.

However, a large number of validation tests are required. The systems pharmacology results can directly prove the effect of the active compounds of SLBZS on the target, while a change in the microbiota structure can supplement the interaction between the Chinese herbal compounds and gut commensals, then revealing the underlying mechanism. Taken together, the combination of the two methods can systematically illustrate the relationship among active compounds, targets, and the microbiota and contribute to the study of the mechanism of IBD.

## 4. Materials and Methods

### 4.1. Dataset Construction

The current data were obtained from the TCM pharmacology analysis platform (TCMSP, http://lsp.nwu.edu.cn/tcmsp.php) and a large amount of literature mining. We collected 980 compounds and their physicochemical properties from SLBZS: 14 compounds of *Dolicho LablabL*, 55 compounds of *Atractylodes macrocephala*, 34 compounds of *Wolfiporia cocos*, 280 compounds of *Glycyrrhiza uralensis Fisch*, 102 compounds of *Platycodon grandiflorus*, 31 compounds of *Semen Nelumbinis*, 190 compounds of *Panax Ginseng*, 165 compounds of *Fructus Amomi*, 71 compounds of *Dioscorea opposita*, and 38 compounds of Semen *Coicis*. Detailed information is freely available from the TCMSP analysis platform.

### 4.2. Active Compound Screening Model

#### 4.2.1. Oral Bioavailability (OB)

In the present study, oral bioavailability (OB) was estimated based on OBioavail 1.1 [85] and the IntegOB. OB is one of the major pharmacokinetic parameters in the ADME (absorption, distribution, metabolism, excretion) profile of a drug. The molecule with suitable OB ≥ 30% served as the candidate compound for further research.

#### 4.2.2. Caco-2 Permeability

The oral absorption of drugs was primarily completed via intestinal epithelial cells (IEC). In this study, a computer Caco-2 permeability prediction model was applied to predict intestinal permeability of all TCM components in TCMSP. The number of Caco − 2 molecules>−0.4 is considered to exhibit adequate intestinal epithelial permeability.

#### 4.2.3. DL (Drug-Likeness)

Drug-likeness is a qualitative concept used in drug design for how “druglike” a substance is with respect to factors like bioavailability. It is estimated from the molecular structure before the substance is even synthesized and tested.

#### 4.2.4. HL (Half-Life)

The half-life of a substance is the duration required for a drug to lose half of its pharmacological activity. HL = long is defined as a suitable half-life range.

### 4.3. Drug Targeting

Drug targeting was implemented by a novel computational model designed to detect the direct drug targets based on an in-house weighted ensemble similarity (WES) method [86] with satisfactory validation of both internal and external data.

### 4.4. Network Construction

TCM is a complex material system comprised of the effective active compound, the target of the action, and the related diseases; it does not correspond to the theory of “single gene, single target, and single disease.” In order to resolve these issues, we constructed a compound-target network to reveal the association between the drug and the target protein. The construction of the network aided in identifying the protein targets of each compound in Chinese medicine, understanding the mechanism underlying the activity of the drug in the treatment of the disease, and studying the target in the disease network. Thus, in the generated network, nodes represent compounds, targets, signaling pathways, or diseases, while edges represent compound-target, target-disease, and target-pathway interactions. The bipartite graphs were generated using Cytoscape 2.8.1 [87].

### 4.5. Compound Organ Location

In order to elucidate the gut disorder at the organ level, firstly, the GO targets were analyzed for most of the selected targets, followed by the analysis of distribution in tissues and organs. Determination of target tissue distribution was based on microarray analysis data of different tissue types in BioGPS database (http://biogps.org).

(1) $$\begin{array}{c} \Omega =\left\{t_{1},t_{2},\cdots ,t_{n}\right\},\\ H_{i}=\left(h_{{it}_{1}},h_{{it}_{2}},\cdots ,h_{{it}_{n}}\right),\\ \bar{h_{i}}=\frac{\sum_{i=1}^{n}h_{i}}{n},\\ A_{i}=\left\{t\in \Omega \;|\;t>\bar{h_{i}}\right\}, \end{array}$$

wherein *t* represents human tissue, *Ω* represents tissue location, *h* represents a tissue-specific pattern of mRNA expression of one target, *H*_*i*_ represents the expression position of mRNA of one target in *Ω*, $\bar{h_{i}}$ represents one target in the tissue of the average expression, *n* is the number of organizations, and *A*_*i*_ indicates a target tissue location.

### 4.6. Animal Management

A rat model test was performed to test the effect of SLBZS on the IBD model. After a week of acclimation period, 30 rats (male and 10 weeks) were randomly divided into 3 groups—control group (CON), model group (MOD), and SLBZS group (SLBZS). The MOD and SLBZS group rats were administered 3% DSS (40 kDa; MP Biomedicals) for 7 days, while the CON was administered with equal volume of saline. After 7 days, the SLBZS group was intragastrically administered with SLBZS 2 mL (1.2 g) for 7 days, and the other two groups were orally given an equal volume of saline. The condition of the rats was monitored twice per day. After 24 hours of the last administration, feces, serum, and colon were collected and stored at -80°C. The animal experiments were approved by the Institutional Animal Care and Use Committee of South China Agricultural University (Approval No. CNAS BL0011).

### 4.7. Preparation of SLBZS

SLBZS was prepared using the published method [7]. SLBZS is a traditional Chinese patent medicine, composed of *Panax Ginseng* (10 g), *Wolfiporia cocos* (10 g), *Atractylodes macrocephala* (10 g), *Dioscorea opposita* (10 g), *Dolichos Lablab* (7.5 g), *Semen Nelumbinis* (5 g), *Semen Coicis* (5 g), *Fructus Amomi* (5 g), *Platycodon grandiflorus* (5 g), and *Glycyrrhiza uralensis Fisch* (5 g). The ten Chinese herbs were purchased from qualified suppliers based on standards specified in the Chinese Pharmacopoeia (Guangzhou, China). The herb materials were mixed and extracted twice at 80°C by stirring it for 1 h using 10 vols of distilled water (v/m). Then, we centrifuged the extract at 1500 ×*g* at room temperature. The SLBZS decoction was filtered and then concentrated to 1 g mL^−1^ (net content) with deionized water.

### 4.8. 16S rRNA Gene Sequence Analysis of Intestinal Flora in Fecal Samples

Total genomic DNA of fecal samples was extracted by the InviMag Stool DNA Kit (Invitek, Germany) as previously described [7]. Fecal microbial DNA was extracted using Fast DNA SPIN extraction kits (MP Biomedicals, Santa Ana, CA, USA) and applied to amplification of the V3-V4 region of 16S rDNA. Fecal microbiota composition was assessed using Illumina HiSeq sequencing of 16S rDNA Amplicon and QIIME-based microbial analysis. The procedures for fecal microbial DNA extraction, sequencing and library construction, and microbial analysis are described in the supplementary methods.

### 4.9. Histologic Observation of the Colon

Histologic colon samples were prepared as previously described [7]. The colon samples were collected and fixed in 10% formalin, dehydrated with a sequence of ethanol solutions, embedded in paraffin, sliced (4-5 *μ*m), and stained with hematoxylin and eosin (HE), then observed with Olympus BH22 Microscope (Japan).

### 4.10. Measurement of Serum Cytokines

Interleukin- (IL-) 1*β*, IL-10, and tumor necrosis factor-alpha (TNF-*α*) were tested using an ELISA kit (Cusabio, Houston, TX, USA; https://www.cusabio.com/). Indices were tested according to the manufacturer's instructions.

### 4.11. Quantification and Statistical Analysis

Statistical analysis was performed with GraphPad Prism 5 (GraphPad Software). Statistical significance was calculated using Kruskal-Wallis test with Dunn's multiple comparison correction. ^∗^ *P* < 0.05, ^∗∗^ *P* < 0.01, and ^∗∗∗^ *P* < 0.001 were regarded as statistically significant.

## Figures and Tables

**Table 1:** Candidate information.

No.	Compound	Herb	OB	Caco-2	HL	DL	Degree	Structure
MOL1	Luteolin	Platycodon grandiflorus Semen Nelumbinis	36.16	0.19	Long	0.25	17	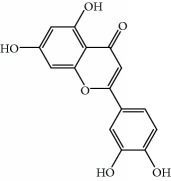

MOL2	12-Senecioyl-2E,8E,10E-atractylentriol	Atractylodes macrocephala	62.40	0.01	Short	0.22	2	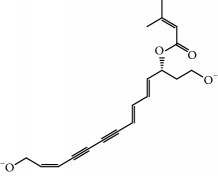

MOL3	14-Acetyl-12-senecioyl-2E,8E,10E-atractylentriol	Atractylodes macrocephala	60.31	0.33	Short	0.31	1	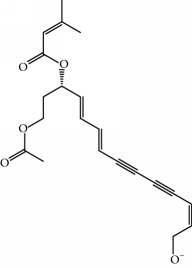

MOL4	14-Acetyl-12-senecioyl-2E,8Z,10E-atractylentriol	Atractylodes macrocephala	63.37	0.42	Short	0.30	1	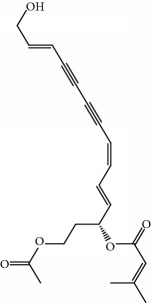

MOL5	Alpha-humulene	Atractylodes macrocephala	22.98	1.88	Short	0.06	1	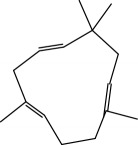

MOL6	(3S,8S,9S,10R,13R,14S,17R)-10,13-Dimethyl-17-[(2R,5S)-5-propan-2-yloctan-2-yl]-2,3,4,7,8,9,11,12,14,15,16,17-dodecahydro-1H-cyclopenta[a]phenanthren-3-ol	Atractylodes macrocephala	36.23	1.45	Long	0.78	3	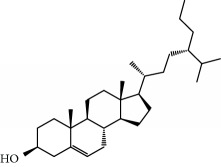

MOL7	Atractylenolide I	Atractylodes macrocephala	37.37	1.30	Long	0.15	2	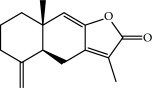

MOL8	3*β*-Acetoxyatractylone	Atractylodes macrocephala	54.07	1.13	Long	0.22	2	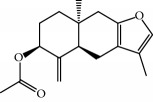

MOL9	Palmitic acid	Dolicho LablabL	19.30	1.09	Short	0.10	4	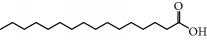

MOL10	Quercetin	Glycyrrhiza uralensis Fisch Semen Nelumbinis	46.43	0.05	Short	0.28	28	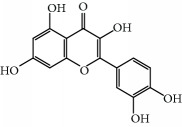

MOL11	Linoleic acid	Dolicho LablabL	41.90	1.16	Short	0.14	3	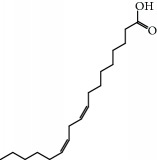

MOL12	(2R)-2-[(3S,5R,10S,13R,14R,16R,17R)-3,16-Dihydroxy-4,4,10,13,14-pentamethyl-2,3,5,6,12,15,16,17-octahydro-1H-cyclopenta[a]phenanthren-17-yl]-6-methylhept-5-enoic acid	Wolfiporia cocos	30.93	0.01	Short	0.81	4	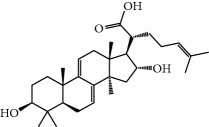

MOL13	Trametenolic acid	Wolfiporia cocos	38.71	0.52	Short	0.80	2	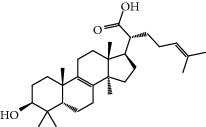

MOL14	7,9(11)-Dehydropachymic acid	Wolfiporia cocos	35.11	0.03	Short	0.81	7	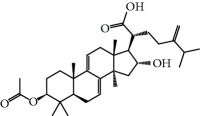

MOL15	Cerevisterol	Wolfiporia cocos	37.96	0.28	Long	0.77	2	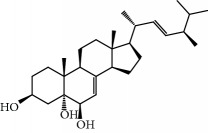

MOL16	(2R)-2-[(3S,5R,10S,13R,14R,16R,17R)-3,16-Dihydroxy-4,4,10,13,14-pentamethyl-2,3,5,6,12,15,16,17-octahydro-1H-cyclopenta[a]phenanthren-17-yl]-5-isopropyl-hex-5-enoic acid	Wolfiporia cocos	31.07	0.05	Short	0.82	5	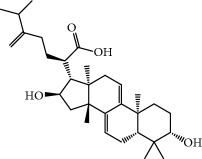

MOL17	Ergosta-7,22E-dien-3beta-ol	Wolfiporia cocos	43.51	1.32	Short	0.72	1	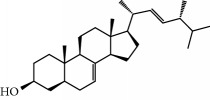

MOL18	(2R)-2-[(5R,10S,13R,14R,16R,17R)-16-Hydroxy-3-keto-4,4,10,13,14-pentamethyl-1,2,5,6,12,15,16,17-octahydrocyclopenta[a]phenanthren-17-yl]-5-isopropyl-hex-5-enoic acid	Wolfiporia cocos	38.26	0.12	Short	0.82	5	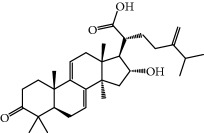

MOL19	3beta-hydroxy-24-methylene-8-lanostene-21-oic acid	Wolfiporia cocos	38.70	0.61	Short	0.81	2	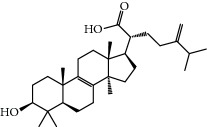

MOL20	Pachymic acid	Wolfiporia cocos	33.63	0.10	Short	0.81	4	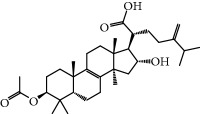

MOL21	Poricoic acid A	Wolfiporia cocos	30.61	-0.14	Short	0.76	5	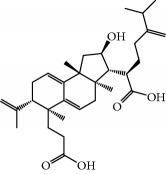

MOL22	Poricoic acid B	Wolfiporia cocos	30.52	-0.08	Short	0.75	4	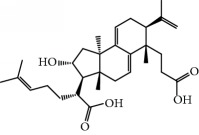

MOL23	Poricoic acid C	Wolfiporia cocos	38.15	0.32	Short	0.75	4	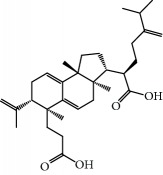

MOL24	Hederagenin	Wolfiporia cocos	36.91	1.32	Short	0.75	3	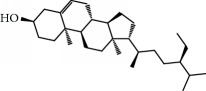

MOL25	Tumulosic acid	Wolfiporia cocos	29.88	0.13	Short	0.81	2	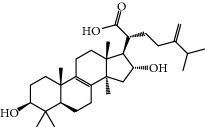

MOL26	Dehydroeburicoic acid	Wolfiporia cocos	44.17	0.38	Short	0.83	3	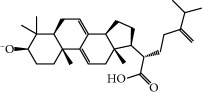

MOL27	Denudatin B	Dioscorea opposita	61.47	0.90	Long	0.38	2	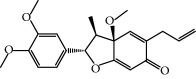

MOL28	Beta-sitosterol	Panax Ginseng Fructus Amomi	36.91	1.32	Short	0.75	3	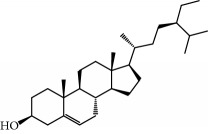

MOL29	Sitosterol	Glycyrrhiza uralensis Fisch Semen Coicis	36.91	1.32	Short	0.75	2	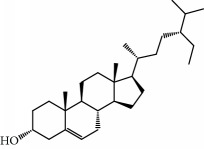

MOL30	Docosanoic acid	Dolicho LablabL	15.69	1.21	Short	0.26	1	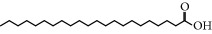

MOL31	Rutin	Glycyrrhiza uralensis Fisch Semen Nelumbinis	3.20	-1.93	Long	0.68	8	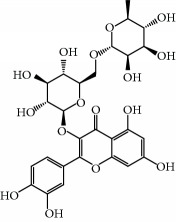

MOL32	Kaempferol	Glycyrrhiza uralensis Fisch Panax Ginseng	41.88	0.26	Long	0.24	15	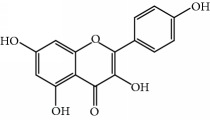

MOL33	Stigmasterol	Panax Ginseng Fructus Amomi Dioscorea opposita Semen Coicis	43.83	1.44	Short	0.76	1	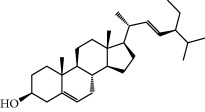

MOL34	Licochalcone A	Glycyrrhiza uralensis Fisch	40.79	0.82	Short	0.29	2	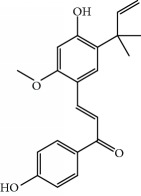

MOL35	Cholesterol	Dioscorea opposita Semen Coicis	37.87	1.43	Short	0.68	3	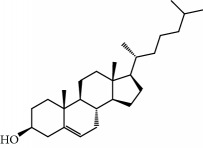

MOL36	Sitosterol alpha1	Semen Coicis	43.28	1.41	Short	0.78	3	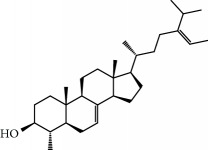

MOL37	Mandenol	Semen Coicis	42.00	1.46	Short	0.19	4	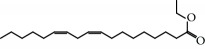

MOL38	24-Ethylcholest-4-en-3-one	Fructus Amomi	36.08	1.46	Short	0.76	1	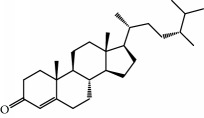

MOL39	Poriferast-5-en-3beta-ol	Fructus Amomi	36.91	1.45	Short	0.75	3	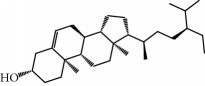

MOL40	Isoliquiritigenin	Glycyrrhiza uralensis Fisch	85.32	0.44	Short	0.15	7	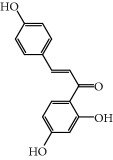

MOL41	Sitosteryl acetate	Fructus Amomi	40.39	1.39	Short	0.85	3	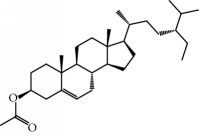

MOL42	[(2R)-2,3-Dihydroxypropyl] (Z)-octadec-9-enoate	Semen Coicis	34.13	0.34	Short	0.30	5	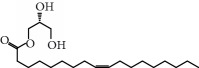

MOL43	Gynesine	Dolicho LablabL	60.07	0.58	Short	0.03	6	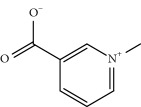

MOL44	Icosa-11,14,17-trienoic acid methyl ester	Fructus Amomi	44.81	1.52	Short	0.23	4	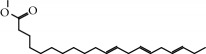

MOL45	Spinasterol	Platycodon grandiflorus	42.98	1.44	Short	0.76	3	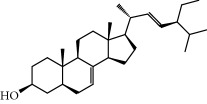

MOL46	Hyperin	Semen Nelumbinis	6.94	-1.42	Short	0.77	4	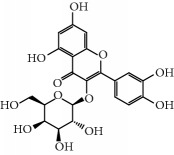

MOL47	18beta-glycyrrhetinic acid	Glycyrrhiza uralensis Fisch	22.05	0.10	Long	0.74	6	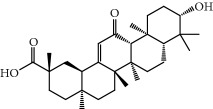

MOL48	Isotrifoliol	Glycyrrhiza uralensis Fisch	31.94	0.53	Long	0.42	3	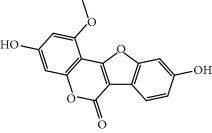

MOL49	(2S)-6-(2,4-Dihydroxyphenyl)-2-(2-hydroxypropan-2-yl)-4-methoxy-2,3-dihydrofuro[3,2-g]chromen-7-one	Glycyrrhiza uralensis Fisch	60.25	0.00	Long	0.63	4	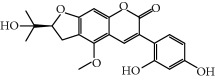

MOL50	Licochalcone B	Glycyrrhiza uralensis Fisch	76.76	0.47	Short	0.19	5	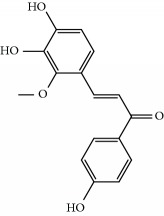

MOL51	Licochalcone C	Glycyrrhiza uralensis Fisch	4.44	0.63	Long	0.29	2	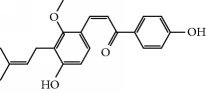

MOL52	Glycyrrhizic acid	Glycyrrhiza uralensis Fisch	19.62	-2.66	Long	0.11	2	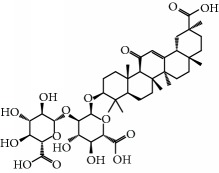

MOL53	Shinpterocarpin	Glycyrrhiza uralensis Fisch	80.30	1.10	Long	0.73	5	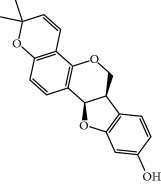

MOL54	Glabridin	Glycyrrhiza uralensis Fisch	53.25	0.97	Long	0.47	5	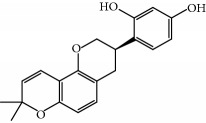

MOL55	Isoglycyrol	Glycyrrhiza uralensis Fisch	44.70	0.91	Long	0.84	1	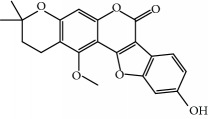

MOL56	Icos-5-enoic acid	Glycyrrhiza uralensis Fisch	30.70	1.22	Short	0.20	3	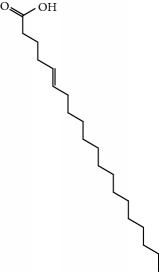

MOL57	Gadelaidic acid	Glycyrrhiza uralensis Fisch	30.70	1.20	Short	0.20	2	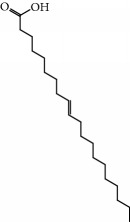

MOL58	Gancaonin H	Glycyrrhiza uralensis Fisch	50.10	0.60	Long	0.78	8	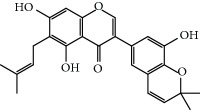

MOL59	18*α*-Hydroxyglycyrrhetic acid	Glycyrrhiza uralensis Fisch	41.16	-0.29	Short	0.71	2	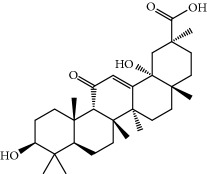

MOL60	Xambioona	Glycyrrhiza uralensis Fisch	54.85	1.09	Long	0.87	3	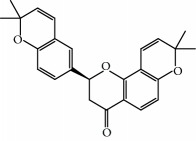

MOL61	Deoxyharringtonine	Panax Ginseng	39.27	0.19	Short	0.81	5	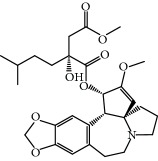

MOL62	Dianthramine	Panax Ginseng	40.45	-0.23	Short	0.20	3	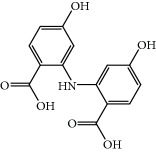

MOL63	Arachidonate	Panax Ginseng	45.57	1.27	Short	0.20	3	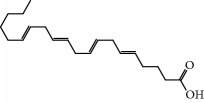

MOL64	Ginsenoside Ro	Panax Ginseng	1.98	-2.86	Long	0.05	3	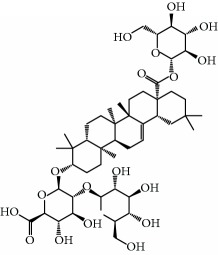

MOL65	Ginsenoside Rb1	Panax Ginseng	6.24	-3.99	Long	0.04	2	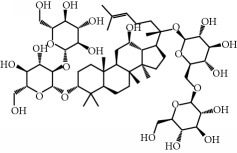

MOL66	Ginsenoside-Rb2	Panax Ginseng	6.02	-3.92	Long	0.04	2	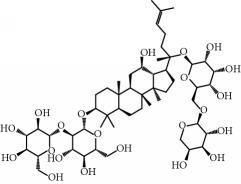

MOL67	Ginsenoside-Rc	Panax Ginseng	8.16	-3.97	Long	0.04	2	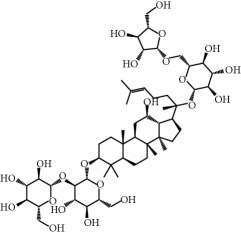

MOL68	Ginsenoside-Rg3	Panax Ginseng	17.75	-2.02	Long	0.22	2	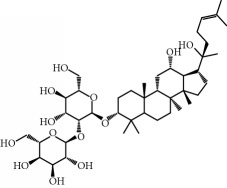

MOL69	Ginsenoside rh2	Panax Ginseng	36.32	-0.51	Long	0.56	2	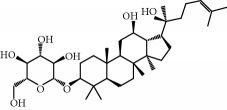

MOL70	Ginsenoside-Rh3_qt	Panax Ginseng	13.09	0.97	Long	0.76	2	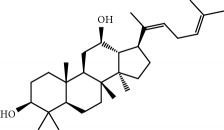

MOL71	Ginsenoside-Rh4	Panax Ginseng	5.22	-0.73	Short	0.60	2	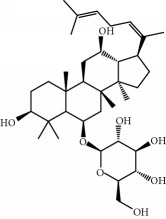

MOL72	Ginsenoside-Rh4_qt	Panax Ginseng	31.11	0.50	Short	0.78	2	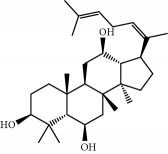

MOL73	Ginsenoside-Rs1	Panax Ginseng	6.27	-3.69	Long	0.04	3	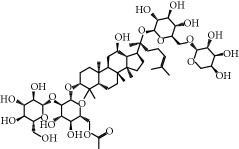

MOL74	Ginsenoside-Rs2	Panax Ginseng	8.14	-4.03	Short	0.04	3	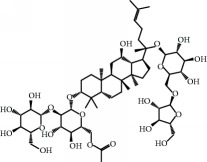

MOL75	Gomisin B	Panax Ginseng	31.99	0.60	Long	0.83	5	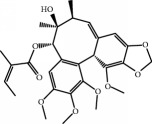

MOL76	Panaxadiol	Panax Ginseng	33.09	0.82	Long	0.79	2	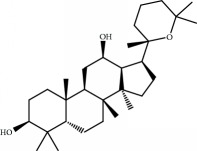

MOL77	Panaxytriol	Panax Ginseng	33.76	0.06	Short	0.13	3	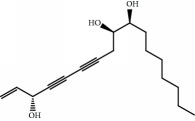

MOL78	Alexandrin_qt	Panax Ginseng	36.91	1.30	Long	0.75	1	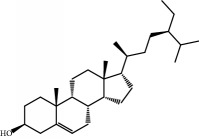

MOL79	Ginsenoside Rg5	Panax Ginseng	6.15	-1.92	Long	0.23	1	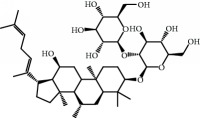

MOL80	Ginsenoside Rg5_qt	Panax Ginseng	39.56	0.88	Long	0.79	2	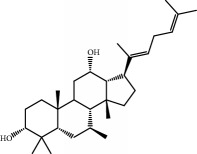

MOL81	Hancinol	Dioscorea opposita	64.01	0.53	Long	0.37	2	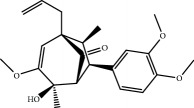

MOL82	Hancinone C	Dioscorea opposita	59.05	0.74	Long	0.39	1	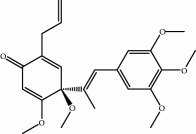

MOL83	24-Methylcholest-5-enyl-3belta-O-glucopyranoside_qt	Dioscorea opposita	37.58	1.33	Short	0.72	1	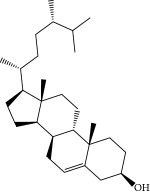

MOL84	Campesterol	Dioscorea opposita	37.58	1.34	Short	0.71	1	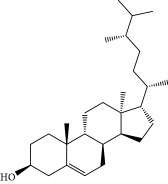

MOL85	Isofucosterol	Dioscorea opposita	43.78	1.36	Short	0.76	1	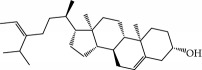

MOL86	Dioscoreside C_qt	Dioscorea opposita	36.38	0.39	Long	0.87	2	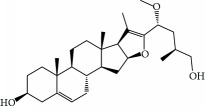

MOL87	Doradexanthin	Dioscorea opposita	38.16	0.52	Short	0.54	4	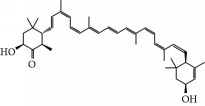

MOL88	Platycodin D	Platycodon grandiflorus	7.60	-4.99	Long	0.01	2	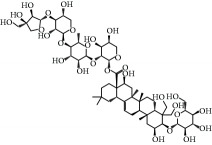

MOL89	Methyl icosa-11,14-dienoate	Fructus Amomi	39.67	1.47	Short	0.23	3	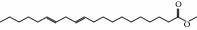

MOL90	(5S,8S,9S,10R,13R,14S,17R)-17-[(1R,4R)-4-Ethyl-1,5-dimethylhexyl]-10,13-dimethyl-2,4,5,7,8,9,11,12,14,15,16,17-dodecahydro-1H-cyclopenta[a]phenanthrene-3,6-dione	Fructus Amomi	33.12	0.90	Short	0.79	1	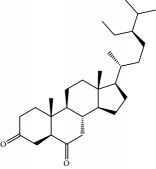

MOL91	Stigmasta-5,22-dien-3-beta-yl acetate	Fructus Amomi	46.44	1.41	Short	0.86	3	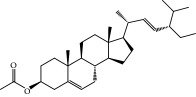

MOL92	Coixenolide	Semen Coicis	32.40	1.09	Short	0.43	1	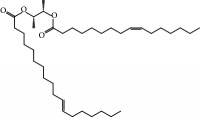

MOL93	2-Monoolein	Semen Coicis	34.23	0.32	Short	0.29	2	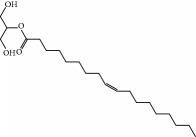

MOL94	Galuteolin	Semen Nelumbinis	2.70	-1.50	Short	0.79	3	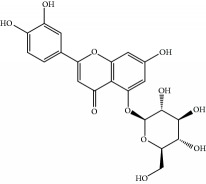

MOL95	14-Methyl-24-methylene-dihydromangiferodiol	Semen Nelumbinis	36.87	0.79	Short	0.80	2	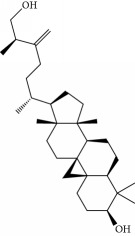

MOL96	4′-Methyl-N-methylcoclaurine	Semen Nelumbinis	55.35	1.44	Long	0.26	2	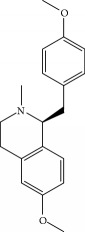

MOL97	Gamabufotalin	Semen Nelumbinis	36.32	-0.29	Short	0.80	1	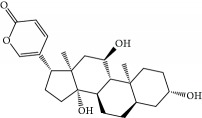

## Data Availability

The data used to support the findings of this study are available from the corresponding authors upon request.

## Abbreviations

IBD: Inflammatory bowel disease
IBS: Irritable bowel syndrome
TCM: Traditional Chinese medicine
IEC: Intestinal epithelial cells
SLBZS: Shen Ling Bai Zhu San
PICRUSt: Phylogenetic investigation of communities by reconstruction of unobserved states
DSS: Dextran sulfate sodium
ADME: Absorption, distribution, metabolism, excretion
OB: Oral bioavailability
Caco-2: Caco-2 permeability
HL: Half-life
DL: Drug-likeness
UPGMA: Unweighted pair-group method with arithmetic.

## Supplementary Materials

Supplementary Materials Tissue location, alteration of phyla and genera, PICRUSt, the information of targets, the relationship between compounds and targets, topology parameters between targets and diseases, the information of pathway, topology parameters between targets and pathway, the relationship between targets and tissues, and supplementary method.
