# Supplementary material for: Systems Pharmacology and Microbiome Dissection of Shen Ling Bai Zhu San Reveal Multiscale Treatment Strategy for IBD
Source: Oxid Med Cell Longev. 2019 Jun 23;2019:8194804. doi: 10.1155/2019/8194804 (PMC6612409; doi:10.1155/2019/8194804)
Supplement: Supplementary Materials — Tissue location, alteration of phyla and genera, PICRUSt, the information of targets, the relationship between compounds and targets, topology parameters between targets and diseases, the information of pathway, topology parameters between targets and pathway, the relationship between targets and tissues, and supplementary method. [file 8194804.f1.zip › Supp Figure S2 Alteration of Phyla and Genera.docx]

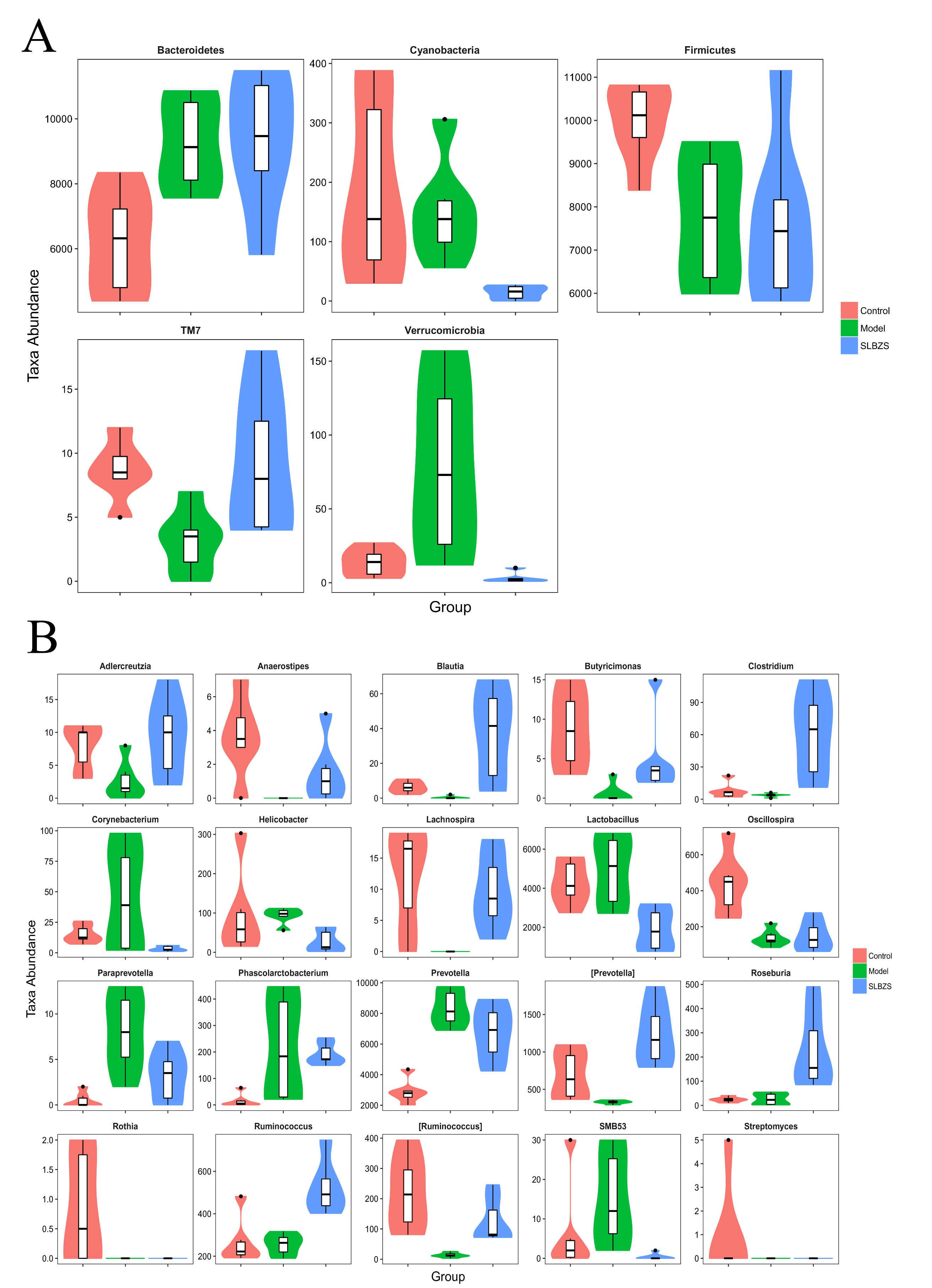


**Supp Fig. S2** SLBZS significantly altered the overall gut microbiota structure in rat model in Phylum (A) and Genus (B) level.
