# Supplementary material for: Systems Pharmacology and Microbiome Dissection of Shen Ling Bai Zhu San Reveal Multiscale Treatment Strategy for IBD
Source: Oxid Med Cell Longev. 2019 Jun 23;2019:8194804. doi: 10.1155/2019/8194804 (PMC6612409; doi:10.1155/2019/8194804)
Supplement: Supplementary Materials — Tissue location, alteration of phyla and genera, PICRUSt, the information of targets, the relationship between compounds and targets, topology parameters between targets and diseases, the information of pathway, topology parameters between targets and pathway, the relationship between targets and tissues, and supplementary method. [file 8194804.f1.zip › Supp Figure S3 PICRUSt.docx]

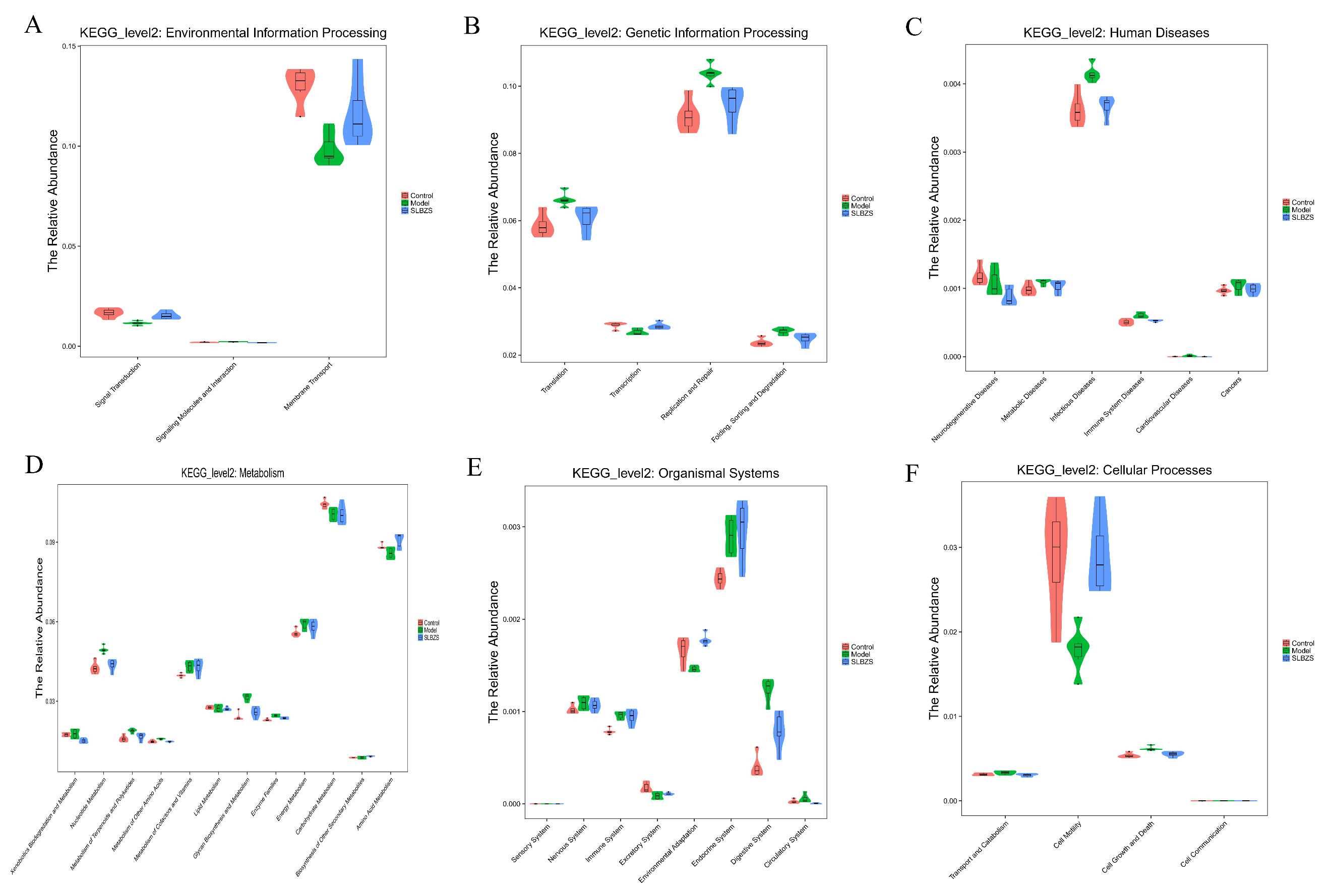


**Supp Fig. S3** KEGG statistics for the functional genes. The horizontal coordinates represent the relative abundance of functional genes in different groups; the vertical coordinates represent the functions of genes. A. Environmental Information Processing. B. Genetic Information Processing. C. Human Diseases. D. Metabolism. E. Organismal Systems. F. Cellular Processes. The relative abundance of functional genes in the following categories was significantly changed (*P*<0.01).
