# Supplementary material for: Systems Pharmacology and Microbiome Dissection of Shen Ling Bai Zhu San Reveal Multiscale Treatment Strategy for IBD
Source: Oxid Med Cell Longev. 2019 Jun 23;2019:8194804. doi: 10.1155/2019/8194804 (PMC6612409; doi:10.1155/2019/8194804)
Supplement: Supplementary Materials — Tissue location, alteration of phyla and genera, PICRUSt, the information of targets, the relationship between compounds and targets, topology parameters between targets and diseases, the information of pathway, topology parameters between targets and pathway, the relationship between targets and tissues, and supplementary method. [file 8194804.f1.zip › Supp Table S1 The information of Targets.docx]

**Supp Table S1 The information of targets**

| **NO.** | **Target-name** | **Gene-Name** | **Degree** |
| --- | --- | --- | --- |
| 1 | Peroxisome proliferator-activated receptor gamma | Pparg | 39 |
| 2 | Sphingosine kinase 1 | Sphk1 | 31 |
| 3 | Krueppel-like factor 5 | Klf5 | 19 |
| 4 | Aldo-keto reductase family 1 member B10 | Akr1b10 | 19 |
| 5 | Lysine-specific demethylase 2A | Kdm2a | 15 |
| 6 | 5-hydroxytryptamine receptor 2A | Htr2a | 15 |
| 7 | Neuronal acetylcholine receptor subunit alpha-4 | Chrna4 | 14 |
| 8 | Neurotensin receptor type 1 | Ntsr1 | 12 |
| 9 | Steryl-sulfatase | Sts | 8 |
| 10 | Arachidonate 5-lipoxygenase | Alox5 | 7 |
| 11 | Secreted frizzled-related protein 1 | Sfrp1 | 7 |
| 12 | Aldehyde dehydrogenase, mitochondrial | Aldh2 | 6 |
| 13 | Tumor necrosis factor receptor superfamily member 1A | Tnfrsf1a | 6 |
| 14 | Bile acid receptor | Nr1h4 | 6 |
| 15 | Prostaglandin E2 receptor EP4 subtype | Ptger4 | 6 |
| 16 | Cytosolic phospholipase A2 | Pla2g4a | 5 |
| 17 | Ornithine decarboxylase | Odc1 | 5 |
| 18 | NADPH oxidase 4 | Nox4 | 4 |
| 19 | Estrogen receptor | Esr1 | 4 |
| 20 | Estrogen receptor beta | Esr2 | 4 |
| 21 | Carbonyl reductase [NADPH] 1 | Cbr1 | 4 |
| 22 | Phospholipase A-2-activating protein | Plaa | 4 |
| 23 | Amine oxidase [flavin-containing] B | Maob | 4 |
| 24 | Prostaglandin E2 receptor EP1 subtype | Ptger1 | 4 |
| 25 | Cytochrome P450 1B1 | Cyp1b1 | 3 |
| 26 | ATP-binding cassette sub-family G member 2 | Abcg2 | 3 |
| 27 | Xanthine dehydrogenase/oxidase [Includes: Xanthine dehydrogenase | Xdh | 3 |
| 28 | Cytochrome P450 1A2 | Cyp1a2 | 3 |
| 29 | Mothers against decapentaplegic homolog 3 | Smad3 | 3 |
| 30 | Kappa-type opioid receptor | Oprk1 | 3 |
| 31 | Protein kinase C beta type | Prkcb | 3 |
| 32 | Proteinase-activated receptor 1 | F2r | 3 |
| 33 | Acid-sensing ion channel 3 | Asic3 | 3 |
| 34 | 72 kDa type IV collagenase | Mmp2 | 2 |
| 35 | G1/S-specific cyclin-E1 | Ccne1 | 2 |
| 36 | Macrophage metalloelastase | Mmp12 | 2 |
| 37 | Matrix metalloproteinase-9 | Mmp9 | 2 |
| 38 | C-C chemokine receptor type 6 | Ccr6 | 2 |
| 39 | Nitric oxide synthase, brain | Nos1 | 2 |
| 40 | Toll-like receptor 2 | Tlr2 | 2 |
| 41 | 5-hydroxytryptamine receptor 1A | Htr1a | 2 |
| 42 | Multidrug resistance-associated protein 1 | Abcc1 | 2 |
| 43 | Coagulation factor VII | F7 | 2 |
| 44 | Heat shock protein beta-1 | Hspb1 | 2 |
| 45 | Integrin alpha-L | Itgal | 2 |
| 46 | Dihydropyrimidine dehydrogenase [NADP(+)] | Dpyd | 2 |
| 47 | Disintegrin and metalloproteinase domain-containing protein 17 | Adam17 | 2 |
| 48 | Poly [ADP-ribose] polymerase 1 | Parp1 | 1 |
| 49 | Proto-oncogene tyrosine-protein kinase Src | Src | 1 |
| 50 | Myeloperoxidase | Mpo | 1 |
| 51 | Prostaglandin G/H synthase 2 | Ptgs2 | 1 |
| 52 | Prolyl endopeptidase | Prep | 1 |
| 53 | Casein kinase II subunit alpha | Csnk2a1 | 1 |
| 54 | C-X-C chemokine receptor type 1 | Cxcr1 | 1 |
| 55 | Sorbitol dehydrogenase | Sord | 1 |
| 56 | Nitric oxide synthase, inducible | Nos2 | 1 |
| 57 | Prostaglandin G/H synthase 1 | Ptgs1 | 1 |
| 58 | DNA polymerase beta | Polb | 1 |
| 59 | Calcitonin gene-related peptide 1 | Calca | 1 |
| 60 | Protein kinase C delta type | Prkcd | 1 |
| 61 | Aryl hydrocarbon receptor | Ahr | 1 |
| 62 | Voltage-dependent calcium channel subunit alpha-2/delta-1 | Cacna2d1 | 1 |
| 63 | Tyrosine-protein phosphatase non-receptor type 2 | Ptpn2 | 1 |
| 64 | Tyrosine-protein phosphatase non-receptor type 11 | Ptpn11 | 1 |
| 65 | Toll-like receptor 4 | Tlr4 | 1 |
| 66 | Vascular endothelial growth factor receptor 1 | Flt1 | 1 |
| 67 | Glutathione S-transferase P 1 | Gstp1 | 1 |
| 68 | Transcription factor p65 | Rela | 1 |
| 69 | Mu-type opioid receptor | Oprm1 | 1 |
| 70 | B1 bradykinin receptor | Bdkrb1 | 1 |
| 71 | Endothelin-1 receptor | Ednra | 1 |
| 72 | DNA-(apurinic or apyrimidinic site) lyase | Apex1 | 1 |
| 73 | Ribonucleoside-diphosphate reductase subunit M2 | Rrm2 | 1 |
| 74 | Insulin-like growth fact or-binding protein 3 | Igfbp3 | 1 |
