# Supplementary material for: Systems Pharmacology and Microbiome Dissection of Shen Ling Bai Zhu San Reveal Multiscale Treatment Strategy for IBD
Source: Oxid Med Cell Longev. 2019 Jun 23;2019:8194804. doi: 10.1155/2019/8194804 (PMC6612409; doi:10.1155/2019/8194804)
Supplement: Supplementary Materials — Tissue location, alteration of phyla and genera, PICRUSt, the information of targets, the relationship between compounds and targets, topology parameters between targets and diseases, the information of pathway, topology parameters between targets and pathway, the relationship between targets and tissues, and supplementary method. [file 8194804.f1.zip › Supp Table S2 The relationship between Compounds and Targets.docx]

**Supp Table S2 The relationship between compounds and targets**

| **NO.** | **Compound** | **Target** |
| --- | --- | --- |
| MOL1 | luteolin | Nox4,Mmp2,Ccne1,Cyp1b1,Mmp12,Mmp9,Abcg2,Xdh,Parp1,Cyp1a2,Aldh2,Tnfrsf1a,Ccr6,Esr1,Esr2,Nos1,Alox5 |
| MOL2 | 12-senecioyl-2E,8E,10E-atractylentriol | Kdm2a,Sphk1 |
| MOL3 | 14-acetyl-12-senecioyl-2E,8E,10E-atractylentriol | Sphk1 |
| MOL4 | 14-acetyl-12-senecioyl-2E,8Z,10E-atractylentriol | Kdm2a |
| MOL5 | alpha-humulene | Chrna4 |
| MOL6 | (3S,8S,9S,10R,13R,14S,17R)-10,13-dimethyl-17-[(2R,5S)-5-propan-2-yloctan-2-yl]-2,3,4,7,8,9,11,12,14,15,16,17-dodecahydro-1H-cyclopenta[a]phenanthren-3-ol | Sphk1,Klf5,Pparg |
| MOL7 | atractylenolide i | Smad3.Chrna4 |
| MOL8 | 3β-acetoxyatractylone | Chrna4,Smad3 |
| MOL9 | palmitic acid | Tlr2,Kdm2a,Sphk1,Klf5 |
| MOL10 | quercetin | Nox4,Oprk1,Htr1a,Mmp2,Ccne1,Src,Cyp1b1,Mpo,Mmp12,Ptgs2,Prep,Alox5,Csnk2a1,Mmp9,Cxcr1,Abcg2,Xdh,Abcc1,Cyp1a2,Sord,Cbr1,Aldh2,Plaa,Maob,Prkcb,Esr2,Esr1,Nos2 |
| MOL11 | Linoleic Acid | F7,Ptgs1,Kdm2a |
| MOL12 | (2R)-2-[(3S,5R,10S,13R,14R,16R,17R)-3,16-dihydroxy-4,4,10,13,14-pentamethyl-2,3,5,6,12,15,16,17-octahydro-1H-cyclopenta[a]phenanthren-17-yl]-6-methylhept-5-enoic acid | Pparg,Akr1b10,Chrna4,Ptger1 |
| MOL13 | trametenolic acid | Pparg.Akr1b10 |
| MOL14 | 7,9(11)-dehydropachymic acid | Pparg,Sphk1,Akr1b10,Nr1h4,Chrna4,Ptger1,Ptger4 |
| MOL15 | Cerevisterol | Pparg,Klf5 |
| MOL16 | (2R)-2-[(3S,5R,10S,13R,14R,16R,17R)-3,16-dihydroxy-4,4,10,13,14-pentamethyl-2,3,5,6,12,15,16,17-octahydro-1H-cyclopenta[a]phenanthren-17-yl]-5-isopropyl-hex-5-enoic acid | Pparg,Akr1b10,Sphk1,Chrna4,Ptger1 |
| MOL17 | ergosta-7,22E-dien-3beta-ol | Pparg |
| MOL18 | (2R)-2-[(5R,10S,13R,14R,16R,17R)-16-hydroxy-3-keto-4,4,10,13,14-pentamethyl-1,2,5,6,12,15,16,17-octahydrocyclopenta[a]phenanthren-17-yl]-5-isopropyl-hex-5-enoic acid | Pparg,Akr1b10,Chrna4,Ptger4,Ptger1 |
| MOL19 | 3beta-Hydroxy-24-methylene-8-lanostene-21-oic acid | Pparg,Akr1b10 |
| MOL20 | pachymic acid | Pparg,Akr1b10,Chrna4,Sphk1 |
| MOL21 | Poricoic acid A | Pparg,Nr1h4,Akr1b10,Chrna4,Ptger4 |
| MOL22 | Poricoic acid B | Pparg,Nr1h4,Akr1b10,Ptger4 |
| MOL23 | poricoic acid C | Pparg,Nr1h4,Akr1b10,Chrna4 |
| MOL24 | hederagenin | Pparg,Klf5,Sphk1 |
| MOL25 | Tumulosic acid | Pparg,Akr1b10 |
| MOL26 | dehydroeburicoic acid | Pparg,Akr1b10,Chrna4 |
| MOL27 | Denudatin B | Sts,Htr2a |
| MOL28 | beta-sitosterol | Polb,Pparg,Klf5 |
| MOL29 | sitosterol | Pparg,Klf5 |
| MOL30 | Docosanoic acid | Kdm2a |
| MOL31 | rutin | Oprk1,Htr1a,Hspb1,Itgal,Calca,Prkcb,Prkcd,F2r |
| MOL32 | kaempferol | Nox4,Cyp1b1,Ahr,Alox5,Abcg2,Xdh,Abcc1,Cyp1a2,Cbr1,Aldh2,Maob,Esr2,Tnfrsf1a,Plaa, Esr1 |
| MOL33 | Stigmasterol | Pparg |
| MOL34 | licochalcone a | Htr2a,Tnfrsf1a |
| MOL35 | Cholesterol | Pparg,Sphk1,Klf5 |
| MOL36 | Sitosterol alpha1 | Sphk1,Klf5,Pparg |
| MOL37 | Mandenol | Sphk1,Kdm2a,Pla2g4a,Klf5 |
| MOL38 | 24-Ethylcholest-4-en-3-one | Pparg |
| MOL39 | poriferast-5-en-3beta-ol | Pparg,Sphk1,Klf5 |
| MOL40 | isoliquiritigenin | Aldh2,Maob,Plaa,Alox5,Dpyd,Tnfrsf1a,Htr2a |
| MOL41 | Sitosteryl acetate | Sphk1,Pparg,Klf5 |
| MOL42 | [(2R)-2,3-dihydroxypropyl] (Z)-octadec-9-enoate | Sphk1,Kdm2a,Klf5,F2r,Pla2g4a |
| MOL43 | gynesine | Kdm2a,Chrna4,Dpyd,Maob,Nos1,Cacna2d1 |
| MOL44 | icosa-11,14,17-trienoic acid methyl ester | Sphk1,Kdm2a,Pla2g4a,Klf5 |
| MOL45 | Spinasterol | Pparg,Klf5,Sphk1 |
| MOL46 | Hyperin | Prkcb,Htr2a,Esr2,Nox4 |
| MOL47 | 18beta-glycyrrhetinic acid | Akr1b10,Ptpn2,Ptpn11,Sfrp1,Pparg,Ptger4 |
| MOL48 | Isotrifoliol | Cbr1,Ccr6,Tlr4 |
| MOL49 | (2S)-6-(2,4-dihydroxyphenyl)-2-(2-hydroxypropan-2-yl)-4-methoxy-2,3-dihydrofuro[3,2-g]chromen-7-one | Htr2a,Klf5,Sts,Flt1 |
| MOL50 | Licochalcone B | Aldh2,Tnfrsf1a,Plaa,Alox5,Htr2a |
| MOL51 | licochalcone C | Htr2a,Tnfrsf1a |
| MOL52 | Glycyrrhizic Acid | Sfrp1,Ntsr1 |
| MOL53 | shinpterocarpin | Odc1,Htr2a,Sts,Aldh2,Asic3 |
| MOL54 | Glabridin | Htr2a,Odc1,Sts,Esr1,Asic3 |
| MOL55 | Isoglycyrol | Sts |
| MOL56 | icos-5-enoic acid | Kdm2a,Sphk1,Klf5 |
| MOL57 | gadelaidic acid | Kdm2a,Sphk1 |
| MOL58 | Gancaonin H | Odc1,F7,Alox5,Gstp1,Rela,Asic3,Htr2a,Sphk1 |
| MOL59 | 18α-hydroxyglycyrrhetic acid | Sfrp1,Ptger4 |
| MOL60 | Xambioona | Odc1,Htr2a,Sts |
| MOL61 | Deoxyharringtonine | Oprk1,Adam17,F2r,Nr1h4,Oprm1 |
| MOL62 | Dianthramine | Cbr1,Smad3,Htr2a |
| MOL63 | arachidonate | Sphk1,Kdm2a,Klf5 |
| MOL64 | ginsenoside Ro | Sfrp1,Sphk1,Ntsr1 |
| MOL65 | ginsenoside Rb1 | Sphk1,Ntsr1 |
| MOL66 | ginsenoside-Rb2 | Sphk1,Ntsr1 |
| MOL67 | ginsenoside-Rc | Sphk1,Ntsr1 |
| MOL68 | Ginsenoside-Rg3 | Ntsr1,Itgal |
| MOL69 | ginsenoside rh2 | Akr1b10,Ntsr1 |
| MOL70 | Ginsenoside-Rh3_qt | Pparg,Akr1b10 |
| MOL71 | Ginsenoside-Rh4 | Nr1h4,Ntsr1 |
| MOL72 | Ginsenoside-Rh4_qt | Pparg,Akr1b10 |
| MOL73 | Ginsenoside-Rs1 | Sphk1,Ntsr1,Sfrp1 |
| MOL74 | Ginsenoside-Rs2 | Ntsr1,Sfrp1,Sphk1 |
| MOL75 | Gomisin B | Adam17,Bdkrb1,Hspb1,Ednra,Apex1 |
| MOL76 | Panaxadiol | Pparg,Akr1b10 |
| MOL77 | Panaxytriol | Kdm2a,Rrm2,Sphk1 |
| MOL78 | alexandrin_qt | Pparg |
| MOL79 | ginsenoside Rg5 | Ntsr1 |
| MOL80 | ginsenoside Rg5_qt | Akr1b10, Pparg |
| MOL81 | hancinol | Sts,Htr2a |
| MOL82 | hancinone C | Odc1 |
| MOL83 | 24-Methylcholest-5-enyl-3belta-O-glucopyranoside_qt | Pparg |
| MOL84 | campesterol | Pparg |
| MOL85 | Isofucosterol | Pparg |
| MOL86 | Dioscoreside C_qt | Pparg, Chrna4 |
| MOL87 | Doradexanthin | Sphk1,Pparg,Pla2g4a,Klf5 |
| MOL88 | Platycodin D | Sfrp1,Ntsr1 |
| MOL89 | methyl icosa-11,14-dienoate | Kdm2a,Pla2g4a,Sphk1 |
| MOL90 | (5S,8S,9S,10R,13R,14S,17R)-17-[(1R,4R)-4-ethyl-1,5-dimethylhexyl]-10,13-dimethyl-2,4,5,7,8,9,11,12,14,15,16,17-dodecahydro-1H-cyclopenta[a]phenanthrene-3,6-dione | Pparg |
| MOL91 | Stigmasta-5,22-dien-3-beta-yl acetate | Sphk1,Pparg,Klf5 |
| MOL92 | Coixenolide | Tlr2 |
| MOL93 | 2-Monoolein | Sphk1,Kdm2a |
| MOL94 | Galuteolin | Htr2a,Alox5,Igfbp3 |
| MOL95 | 14-Methyl-24-methylene-dihydromangiferodiol | Pparg,Akr1b10 |
| MOL96 | 4'-methyl-N-methylcoclaurine | Htr2a,Sts |
| MOL97 | Gamabufotalin | Chrna4 |
