# Supplementary material for: Systems Pharmacology and Microbiome Dissection of Shen Ling Bai Zhu San Reveal Multiscale Treatment Strategy for IBD
Source: Oxid Med Cell Longev. 2019 Jun 23;2019:8194804. doi: 10.1155/2019/8194804 (PMC6612409; doi:10.1155/2019/8194804)
Supplement: Supplementary Materials — Tissue location, alteration of phyla and genera, PICRUSt, the information of targets, the relationship between compounds and targets, topology parameters between targets and diseases, the information of pathway, topology parameters between targets and pathway, the relationship between targets and tissues, and supplementary method. [file 8194804.f1.zip › Supp Table S4 The information of pathway.docx]

**Supp Table S4 The information of pathway**

| **NO.** | **Pathway ID** | **Pathway** |
| --- | --- | --- |
| 1 | mmu04020 | Calcium signaling pathway |
| 2 | mmu05200 | Pathways in cancer |
| 3 | mmu04750 | Inflammatory mediator regulation of TRP channels |
| 4 | mmu04726 | Serotonergic synapse |
| 5 | mmu04080 | Neuroactive ligand-receptor interaction |
| 6 | mmu04370 | VEGF signaling pathway |
| 7 | mmu04915 | Estrogen signaling pathway |
| 8 | mmu00330 | Arginine and proline metabolism |
| 9 | mmu05161 | Hepatitis B |
| 10 | mmu05205 | Proteoglycans in cancer |
| 11 | mmu05142 | Chagas disease (American trypanosomiasis) |
| 12 | mmu05140 | Leishmaniasis |
| 13 | mmu05145 | Toxoplasmosis |
| 14 | mmu05146 | Amoebiasis |
| 15 | mmu05152 | Tuberculosis |
| 16 | mmu04912 | GnRH signaling pathway |
| 17 | mmu00590 | Arachidonic acid metabolism |
| 18 | mmu00380 | Tryptophan metabolism |
| 19 | mmu05204 | Chemical carcinogenesis |
| 20 | mmu04064 | NF-kappa B signaling pathway |
| 21 | mmu04066 | HIF-1 signaling pathway |
| 22 | mmu04913 | Ovarian steroidogenesis |
| 23 | mmu05202 | Transcriptional misregulation in cancer |
| 24 | mmu05321 | Inflammatory bowel disease (IBD) |
| 25 | mmu00980 | Metabolism of xenobiotics by cytochrome P450 |
| 26 | mmu04670 | Leukocyte transendothelial migration |
| 27 | mmu04071 | Sphingolipid signaling pathway |
| 28 | mmu04917 | Prolactin signaling pathway |
| 29 | mmu05162 | Measles |
| 30 | mmu05323 | Rheumatoid arthritis |
| 31 | mmu05222 | Small cell lung cancer |
| 32 | mmu03410 | Base excision repair |
| 33 | mmu00040 | Pentose and glucuronate interconversions |
| 34 | mmu04921 | Oxytocin signaling pathway |
| 35 | mmu05219 | Bladder cancer |
| 36 | mmu00232 | Caffeine metabolism |
| 37 | mmu05144 | Malaria |
| 38 | mmu04010 | MAPK signaling pathway |
| 39 | mmu04668 | TNF signaling pathway |
| 40 | mmu04931 | Insulin resistance |
| 41 | mmu01100 | Metabolic pathways |
| 42 | mmu00480 | Glutathione metabolism |
| 43 | mmu05134 | Legionellosis |
| 44 | mmu04062 | Chemokine signaling pathway |
| 45 | mmu05206 | MicroRNAs in cancer |
| 46 | mmu04270 | Vascular smooth muscle contraction |
| 47 | mmu04730 | Long-term depression |
