# Supplementary material for: Systems Pharmacology and Microbiome Dissection of Shen Ling Bai Zhu San Reveal Multiscale Treatment Strategy for IBD
Source: Oxid Med Cell Longev. 2019 Jun 23;2019:8194804. doi: 10.1155/2019/8194804 (PMC6612409; doi:10.1155/2019/8194804)
Supplement: Supplementary Materials — Tissue location, alteration of phyla and genera, PICRUSt, the information of targets, the relationship between compounds and targets, topology parameters between targets and diseases, the information of pathway, topology parameters between targets and pathway, the relationship between targets and tissues, and supplementary method. [file 8194804.f1.zip › Supplementary Method.docx]

**Supplementary Methods**

**DNA Extraction**

Total bacterial genomic DNA samples were extracted using the Fast DNA SPIN extraction kits (MP Biomedicals, Santa Ana, CA, USA), following the manufacturer’s instructions, and stored at −20°C prior to further analysis. The quantity and quality of extracted DNAs were measured using a NanoDrop ND-1000 spectrophotometer (Thermo Fisher Scientific, Waltham, MA,USA) and agarose gel electrophoresis, respectively.

**16S rDNA Amplicon Pyrosequencing**

PCR amplification of the bacterial 16S rRNA genes V3–V4 region was performed using the forward primer 338F (5’- ACTCCTACGGGAGGCAGCA-3’) and the reverse primer 806R (5’- GGACTACHVGGGTWTCTAAT-3’). Sample-specific 7-bp barcodes were incorporated into the primers for multiplex sequencing. The PCR components contained 5 μl of Q5 reaction buffer (5×), 5 μl of Q5 High-Fidelity GC buffer (5×), 0.25 μl of Q5 High-Fidelity DNA Polymerase (5U/μl), 2 μl (2.5 mM) of dNTPs, 1 μl (10 uM) of each Forward and Reverse primer, 2 μl of DNA Template, and 8.75 μl of ddH2O. Thermal cycling consisted of initial denaturation at 98 °C for 2 min, followed by 25 cycles consisting of denaturation at 98 °C for 15 s, annealing at 55 °C for 30 s, and extension at 72 °C for 30 s, with a final extension of 5 min at 72 °C. PCR amplicons were purified with Agencourt AMPure Beads (Beckman Coulter, Indianapolis, IN) and quantified using the PicoGreen dsDNA Assay Kit (Invitrogen, Carlsbad, CA, USA). After the individual quantification step, amplicons were pooled in equal amounts, and pair-end 2×300 bp sequencing was performed using the Illlumina MiSeq platform with MiSeq Reagent Kit v3 at Shanghai Personal Biotechnology Co., Ltd (Shanghai, China).

**Sequence Analysis**

The Quantitative Insights Into Microbial Ecology (QIIME, v1.8.0) pipeline was employed to process the sequencing data, as previously described (Caporaso, Kuczynski et al. 2010). Briefly, raw sequencing reads with exact matches to the barcodes were assigned to respective samples and identified as valid sequences. The low-quality sequences were filtered through following criteria (Gill, Pop et al. 2006, Chen and Jiang 2014): sequences that had a length of <150 bp, sequences that had average Phred scores of <20, sequences that contained ambiguous bases, and sequences that contained mononucleotide repeats of >8 bp. Paired-end reads were assembled using FLASH (Magoc and Salzberg 2011). After chimera detection, the remaining high-quality sequences were clustered into operational taxonomic units (OTUs) at 97% sequence identity by UCLUST (Edgar 2010). A representative sequence was selected from each OTU using default parameters. OTU taxonomic classification was conducted by BLAST searching the representative sequences set against the Greengenes Database (DeSantis, Hugenholtz et al. 2006) using the best hit (Altschul, Madden et al. 1997). An OTU table was further generated to record the abundance of each OTU in each sample and the taxonomy of these OTUs. OTUs containing less than 0.001% of total sequences across all samples were discarded. To minimize the difference of sequencing depth across samples, an averaged, rounded rarefied OTU table was generated by averaging 100 evenly resampled OTU subsets under the 90% of the minimum sequencing depth for further analysis.

**Bioinformatics and Statistical Analysis**

Sequence data analyses were mainly performed using QIIME and R packages (v3.2.0). OTU-level alpha diversity indices, such as Chao1 richness estimator, ACE metric (Abundance-based Coverage Estimator), Shannon diversity index, and Simpson index, were calculated using the OTU table in QIIME. OTU-level ranked abundance curves were generated to compare the richness and evenness of OTUs among samples. Beta diversity analysis was performed to investigate the structural variation of microbial communities across samples using UniFrac distance metrics (Lozupone and Knight 2005, Lozupone, Hamady et al. 2007) and visualized via principal coordinate analysis (PCoA), nonmetric multidimensional scaling (NMDS) and unweighted pair-group method with arithmetic means (UPGMA) hierarchical clustering (Ramette 2007). Differences in the Unifrac distances for pairwise comparisons among groups were determined using Student’s t-test and the Monte Carlo permutation test with 1000 permutations, and visualized through the box-and-whiskers plots. Principal component analysis (PCA) was also conducted based on the genus-level compositional profiles (Ramette 2007). The significance of differentiation of microbiota structure among groups was assessed by PERMANOVA (Permutational multivariate analysis of variance) (McArdle and Anderson 2001) and ANOSIM (Analysis of similarities) (Clarke 1993, Warton, Wright et al. 2012) using R package “vegan”. The taxonomy compositions and abundances were visualized using MEGAN (Huson, Mitra et al. 2011) and GraPhlAn (Asnicar, Weingart et al. 2015). Venn diagram was generated to visualize the shared and unique OTUs among samples or groups using R package “VennDiagram”, based on the occurrence of OTUs across samples/groups regardless of their relative abundance (Zaura, Keijser et al. 2009). Taxa abundances at the phylum, class, order, family, genus and species levels were statistically compared among samples or groups by Metastats (White, Nagarajan et al. 2009), and visualized as violin plots. LEfSe (Linear discriminant analysis effect size) was performed to detect differentially abundant taxa across groups using the default parameters (Segata, Izard et al. 2011). PLS-DA (Partial least squares discriminant analysis) was also introduced as a supervised model to reveal the microbiota variation among groups, using the “plsda” function in R package “mixOmics” (Chen, Yang et al. 2011). Random forest analysis was applied to discriminating the samples from different groups using the R package “randomForest” with 1,000 trees and all default settings (Breiman 2001, Liaw and Wiener 2002). The generalization error was estimated using 10-fold cross-validation. The expected “baseline” error was also included, which was obtained by a classifier that simply predicts the most common category label. Co-occurrence analysis was performed by calculating Spearman’s rank correlations between predominant taxa. Correlations with |RHO| > 0.6 and *P* < 0.01 were visualized as co-occurrence network using Cytoscape (Shannon, Markiel et al. 2003). Microbial functions were predicted by PICRUSt (Phylogenetic investigation of communities by reconstruction of unobserved states), based on high-quality sequences (Langille, Zaneveld et al. 2013).

**References**

Altschul, S. F., T. L. Madden, A. A. Schaffer, J. H. Zhang, Z. Zhang, W. Miller and D. J. Lipman (1997). "Gapped BLAST and PSI-BLAST: a new generation of protein database search programs." Nucleic Acids Research **25**(17): 3389-3402.

Asnicar, F., G. Weingart, T. L. Tickle, C. Huttenhower and N. Segata (2015). "Compact graphical representation of phylogenetic data and metadata with GraPhlAn." Peerj **3**.

Breiman, L. (2001). "Random forests." Machine Learning **45**(1): 5-32.

Caporaso, J. G., J. Kuczynski, J. Stombaugh, K. Bittinger, F. D. Bushman, E. K. Costello, N. Fierer, A. G. Pena, J. K. Goodrich, J. I. Gordon, G. A. Huttley, S. T. Kelley, D. Knights, J. E. Koenig, R. E. Ley, C. A. Lozupone, D. McDonald, B. D. Muegge, M. Pirrung, J. Reeder, J. R. Sevinsky, P. J. Tumbaugh, W. A. Walters, J. Widmann, T. Yatsunenko, J. Zaneveld and R. Knight (2010). "QIIME allows analysis of high-throughput community sequencing data." Nature Methods **7**(5): 335-336.

Chen, H. and W. Jiang (2014). "Application of high-throughput sequencing in understanding human oral microbiome related with health and disease." Frontiers in Microbiology **5**: 6.

Chen, Y. F., F. L. Yang, H. F. Lu, B. H. Wang, Y. B. Chen, D. J. Lei, Y. Z. Wang, B. L. Zhu and L. J. Li (2011). "Characterization of Fecal Microbial Communities in Patients with Liver Cirrhosis." Hepatology **54**(2): 562-572.

Clarke, K. R. (1993). "Non-parametric multivariate analyses of changes in community structure." Australian Journal of Ecology **18**(1): 117-143.

DeSantis, T. Z., P. Hugenholtz, N. Larsen, M. Rojas, E. L. Brodie, K. Keller, T. Huber, D. Dalevi, P. Hu and G. L. Andersen (2006). "Greengenes, a chimera-checked 16S rRNA gene database and workbench compatible with ARB." Applied and environmental microbiology **72**(7): 5069-5072.

Edgar, R. C. (2010). "Search and clustering orders of magnitude faster than BLAST." Bioinformatics **26**(19): 2460-2461.

Gill, S. R., M. Pop, R. T. DeBoy, P. B. Eckburg, P. J. Turnbaugh, B. S. Samuel, J. I. Gordon, D. A. Relman, C. M. Fraser-Liggett and K. E. Nelson (2006). "Metagenomic analysis of the human distal gut microbiome." Science **312**(5778): 1355-1359.

Huson, D. H., S. Mitra, H.-J. Ruscheweyh, N. Weber and S. C. Schuster (2011). "Integrative analysis of environmental sequences using MEGAN4." Genome Research **21**(9): 1552-1560.

Langille, M. G. I., J. Zaneveld, J. G. Caporaso, D. McDonald, D. Knights, J. A. Reyes, J. C. Clemente, D. E. Burkepile, R. L. V. Thurber, R. Knight, R. G. Beiko and C. Huttenhower (2013). "Predictive functional profiling of microbial communities using 16S rRNA marker gene sequences." Nature Biotechnology **31**(9): 814-+.

Liaw, A. and M. Wiener (2002). "Classification and regression by randomForest." R News **2**(3): 18-22.

Lozupone, C. and R. Knight (2005). "UniFrac: a new phylogenetic method for comparing microbial communities." Applied and environmental microbiology **71**(12): 8228-8235.

Lozupone, C. A., M. Hamady, S. T. Kelley and R. Knight (2007). "Quantitative and qualitative beta diversity measures lead to different insights into factors that structure microbial communities." Applied and Environmental Microbiology **73**(5): 1576-1585.

Magoc, T. and S. L. Salzberg (2011). "FLASH: fast length adjustment of short reads to improve genome assemblies." Bioinformatics **27**(21): 2957-2963.

McArdle, B. H. and M. J. Anderson (2001). "Fitting multivariate models to community data: a comment on distance-based redundancy analysis." Ecology **82**(1): 290-297.

Ramette, A. (2007). "Multivariate analyses in microbial ecology." Fems Microbiology Ecology **62**(2): 142-160.

Segata, N., J. Izard, L. Waldron, D. Gevers, L. Miropolsky, W. S. Garrett and C. Huttenhower (2011). "Metagenomic biomarker discovery and explanation." Genome Biology **12**(6).

Shannon, P., A. Markiel, O. Ozier, N. S. Baliga, J. T. Wang, D. Ramage, N. Amin, B. Schwikowski and T. Ideker (2003). "Cytoscape: A software environment for integrated models of biomolecular interaction networks." Genome Research **13**(11): 2498-2504.

Warton, D. I., S. T. Wright and Y. Wang (2012). "Distance-based multivariate analyses confound location and dispersion effects." Methods in Ecology and Evolution **3**(1): 89-101.

White, J. R., N. Nagarajan and M. Pop (2009). "Statistical Methods for Detecting Differentially Abundant Features in Clinical Metagenomic Samples." Plos Computational Biology **5**(4).

Zaura, E., B. J. F. Keijser, S. M. Huse and W. Crielaard (2009). "Defining the healthy "core microbiome" of oral microbial communities." Bmc Microbiology **9**: 12.
